# Impacts of combining anti-PD-L1 immunotherapy and radiotherapy on the tumour immune microenvironment in a murine prostate cancer model

**DOI:** 10.1038/s41416-020-0956-x

**Published:** 2020-07-09

**Authors:** Yiannis Philippou, Hanna T. Sjoberg, Emma Murphy, Said Alyacoubi, Keaton I. Jones, Alex N. Gordon-Weeks, Su Phyu, Eileen E. Parkes, W. Gillies McKenna, Alastair D. Lamb, Uzi Gileadi, Vincenzo Cerundolo, David A. Scheiblin, Stephen J. Lockett, David A. Wink, Ian G. Mills, Freddie C. Hamdy, Ruth J. Muschel, Richard J. Bryant

**Affiliations:** 1 Department of Oncology, University of Oxford, Oxford, UK; 2 Nuffield Department of Surgical Sciences, University of Oxford, Oxford, UK; 3 MRC Human Immunology Unit, Weatherall Institute of Molecular Medicine, University of Oxford, Oxford, UK; 4 Optical Microscopy and Analysis Laboratory, Frederick National Laboratory for Cancer Research, Leidos Biomedical Research Inc. for the National Cancer Institute, National Institutes of Health, Frederick, 21702 MD USA; 5 Cancer and Inflammation Program, Center for Cancer Research, National Cancer Institute, National Institutes of Health, Frederick, 21702 MD USA

**Keywords:** Prostate cancer, Cancer microenvironment

## Abstract

**Background:**

Radiotherapy enhances innate and adaptive anti-tumour immunity. It is unclear whether this effect may be harnessed by combining immunotherapy with radiotherapy fractions used to treat prostate cancer. We investigated tumour immune microenvironment responses of pre-clinical prostate cancer models to radiotherapy. Having defined this landscape, we tested whether radiotherapy-induced tumour growth delay could be enhanced with anti-PD-L1.

**Methods:**

Hypofractionated radiotherapy was delivered to TRAMP-C1 and MyC-CaP flank allografts. Tumour growth delay, tumour immune microenvironment flow-cytometry, and immune gene expression were analysed. TRAMP-C1 allografts were then treated with 3 × 5 Gy ± anti-PD-L1.

**Results:**

3 × 5 Gy caused tumour growth delay in TRAMP-C1 and MyC-CaP. Tumour immune microenvironment changes in TRAMP-C1 at 7 days post-radiotherapy included increased tumour-associated macrophages and dendritic cells and upregulation of PD-1/PD-L1, CD8^+^ T-cell, dendritic cell, and regulatory T-cell genes. At tumour regrowth post-3 × 5 Gy the tumour immune microenvironment flow-cytometry was similar to control tumours, however CD8^+^, natural killer and dendritic cell gene transcripts were reduced. PD-L1 inhibition plus 3 × 5 Gy in TRAMP-C1 did not enhance tumour growth delay versus monotherapy.

**Conclusion:**

3 × 5 Gy hypofractionated radiotherapy can result in tumour growth delay and immune cell changes in allograft prostate cancer models. Adjuncts beyond immunomodulation may be necessary to improve the radiotherapy-induced anti-tumour response.

## Background

Radiotherapy (RT) combined with androgen deprivation therapy (ADT) is a standard-of-care therapy for high risk localised or locally advanced prostate cancer (PCa).^1,2^ Technological advances in RT delivery have improved treatment efficacy, however patients with high-risk PCa have a 32–70% 5-year biochemical recurrence rate,^3^ and RT reduces the quality of life due to side effects.^4–6^ Improved RT efficacy in terms of cure, and reduced treatment toxicity through the use of reduced RT doses, might be achieved by combining immunomodulation with RT (iRT). RT to the primary tumour in low-burden metastatic PCa may also provide benefit^7^ through RT-induced anti-tumour immune responses enhanced with iRT. Whilst iRT may improve tumour control, the evidence from pre-clinical PCa models investigating RT combined with adjuncts^8–14^ frequently use RT doses considerably higher than in clinical practice, such as 10, 20, or 25 Gy fractions. Standard clinical RT delivers 2 Gy fractions over 6 weeks to a dose of 74–78 Gy. Recent developments have seen moderate hypofractionation with 62 Gy in 20 fractions over 4 weeks, and ultrahypofractionation with 36.25 Gy in 5 fractions over 1–2 weeks, with no increase in acute toxicity.^15^ Although these studies suggest a change in practice with increased dose per fraction, and reduced overall numbers of fractions, the dose per fraction in clinical practice remains lower than those in pre-clinical iRT PCa experiments.

Anti-tumour RT effects include DNA breaks causing apoptosis,^16^ immunological effects^17–19^ and anti-tumour immune responses.^18,20–23^ Despite the observation of pro-inflammatory tumour effects following RT, the immunosuppressive tumour immune microenvironment (TIME) of pre-clinical models includes abundant tumour-associated macrophages (TAMs) and myeloid-derived suppressor cells (MDSCs). These myeloid-derived cells may impede RT-induced anti-tumour responses^24,25^ and promote tumour progression through growth factors, cytokines, chemokines and reactive oxygen species, increasing survival and growth of cancer cells. Although RT is a potential immunostimulatory anti-PCa therapy, with opportunities for combination with immunotherapy, it may be difficult to harness immune-mediated anti-tumour responses in PCa based on its moderate mutational burden and disappointing immunotherapy clinical trial results.^26–28^ However, the first FDA-approved immunotherapy, albeit without RT, for a solid-organ cancer was Sipuleucel-T in PCa,^29–31^ and immunomodulation may be effective if appropriately combined with RT. A clinical study combining anti-CTLA4 with a 8 Gy fraction to PCa bony metastases demonstrated a non-significant improvement in overall survival,^32,33^ and a trial investigating the efficacy of anti-PD-1 and RT in high-risk and oligometastatic PCa is underway.^34^

To test the hypothesis that RT fractions used in the clinic induce PCa TIME effects, flank tumour allografts in immunocompetent mice were treated with RT and effects of RT investigated as a primary objective using tumour growth delay, flow cytometry (FACS) and NanoString immune gene expression changes. Effects of iRT with concomitant anti-PD-L1 were investigated as a secondary objective, testing the hypothesis that iRT increases anti-tumour effects.

## Methods

### Cell lines and cell culture

TRAMP-C1 (ATCC® CRL-2730™) and MyC-CaP (ATCC® CRL-3255™) cells were purchased from American Type Culture Collection (ATCC®). TRAMP-C1 cells were derived from the TRAMP (transgenic adenocarcinoma mouse prostate) model in C57BL/6 mice harbouring a construct comprising the minimal rat probasin promoter driving prostate-specific epithelial expression of SV40 large T antigen.^35,36^ MyC-CaP cells were derived from a *c-myc* transgenic mouse with prostate cancer.^37^ Both TRAMP-C1 and MyC-CaP cells express androgen receptor,^36–38^ however, TRAMP-C1 are androgen-independent whilst MyC-CaP cells are androgen-sensitive. TRAMP-C1 and MyC-CaP cells are useful for immunocompetent subcutaneous tumour models investigating therapies requiring an immune system prior to clinical trials. TRAMP-C1 cells were cultured in Dulbecco’s modified Eagle’s medium (DMEM) with 4 mM L-glutamine, 1.5 g/L sodium bicarbonate, 4.5 g/L glucose, 0.005 mg/ml bovine insulin, 10 nM dehydroisoandrosterone, 5% Nu-Serum IV, 5% foetal bovine serum (FBS), 1% penicillin and 100 μg/ml streptomycin. MyC-CaP cells were cultured in DMEM with 10% FBS, 1% penicillin and 100 μg/ml streptomycin. Cells were maintained mycoplasma free (LookOut® Mycoplasma PCR Detection Kit) at 37 °C and 5% CO_2_.

### FACS and western blot analysis of in vitro MHC-I and PD-L1 expression following IFN-γ and radiotherapy

40,000 TRAMP-C1 or MyC-CaP cells were plated in duplicate in 6-well plates and treated with 0, 0.1, 1.0 or 10 ng/mL mouse recombinant murine gamma interferon (γIFN) (Invitrogen PMC4034) for FACS quantification of MHC-I complex (H-2K^b^) and PD-L1 expression, and changes induced by γIFN. To test whether RT induces MHC-I and PD-L1 expression, TRAMP-C1 cells were treated with 2, 6 and 10 Gy RT using a Caesium irradiator at 1.46 Gy/min ± 1.0 ng/mL γIFN. After 72 h incubation cells were detached using Accutase solution, washed with PBS and stained with Pe-Cy7 H-2Kb (MHC-I, eBioscience, AF6–88.5.53), BV421-CD274 (B7-H1, PD-L1, Biolegend, 10F.9G2) and LIVE/DEAD™ Fixable Green Dead Cell Stain Kit (ThermoFisher Scientific, L23101) or Propidium Iodide in 100 μl FACS buffer (PBS with 2% FBS). For western blot analysis, whole-cell extracts were prepared from PBS-washed cells using RIPA lysis buffer containing protease and phosphatase inhibitors. Protein concentration was determined using Pierce™ Rapid Gold BCA Protein Assay Kit (Thermoscientific) using a bovine serum albumin standard. Proteins were reduced at 95 ^o^C with Laemmli gel loading buffer containing 2-mercaptoethanol, separated on 4–15% polyacrylamide gels containing SDS and transferred to PVDF membranes. Anti-PD-L1 (1:400, R&D systems) and vinculin (1:1000, sc-25336 Santa Cruz Biotechnology) primary antibodies were used, and membranes incubated with 1:5000 HRP conjugated secondary antibodies (anti-Goat, Sigma-Aldrich and anti-Rabbit IgG, GeneTex, respectively) and visualised with ECL detection reagent (Amersham, GE Healthcare).

### Tumour challenge and treatment experiments

Animal procedures were performed according to UK Animal law (Scientific Procedures Act 1986) and ARRIVE guidelines, with local ethics and Home Office approval. Naive male 6–8 week old immunocompetent C57BL/6 and FVB mice (*Mus Musculus*, Charles River Laboratories, UK) were housed in groups of six to limit fighting, in a pathogen-free facility with 12-h light cycles, in individually ventilated cages on woodchip bedding, with access to water and food *ad libitum*, at 22 °C (range 21–24 °C) and 50% humidity (range 35–75%), with environmental enrichment and bedding material, and monitored for body weight changes twice weekly. TRAMP-C1 (2 × 10^6^) or MyC-CaP (1.0 × 10^6^) cells in PBS and 1:1 high concentration phenol red-free Matrigel (Corning) were injected into the flank of mice under isofluorane inhalational anaesthesia on a heat mat to aid recovery. Tumours were measured pre- and post-treatment using digital callipers three times weekly (tumour volume = π/6 × length × width × height). When tumours reached 100 mm^3^ mice were assigned to treatment groups on a ‘first come, first allocated’ basis using a randomly generated treatment list (GraphPad Prism 8, GraphPad Software, USA). RT was delivered in the afternoon under isofluorane inhalational anaesthesia with physiological monitoring (pneumatic cushion; breathing rate 40–60 breaths/min) on a heat mat using a Gulmay 320 irradiator (300 kV, 10 mA, 2.25 Gy/min). 10 mg/kg anti-PD-L1^39^ (BioXCell, clone 10F.9G2) or isotype control (BioXCell, rat IgG2b) in PBS was administered by intra-peritoneal injection on days 1 (i.e. with the first 5 Gy RT fraction at 100 mm^3^ tumour size), 4, 7, and 10.^39^ All procedures were conducted in the home cage except tumour cell injection, RT and recovery from anaesthetic. All mice were culled by a schedule one procedure at end-point in the morning using pentobarbitone injection overdose followed by cervical dislocation according to institutional guidelines.

### Flow cytometry (FACS) analysis of tumours following in vivo treatment with radiotherapy

Tumours were mechanically dissociated into 2–3 mm pieces and treated with 5 mL digestion cocktail containing 400 µl 5 g/ml collagenase IV (Worthington, LS004189), 5 U/mL DNAse I (195 U/mL) (Invitrogen, 1928344) in Hanks Balanced Salt Solution and passed through a 70 µm nylon cell strainer. Red blood cells were removed using lysis buffer (BioLegend, B256521). Cells were incubated for 5 min at room temperature with purified anti-mouse CD16/CD32 Fc block (BioLegend, B255480) before the addition of cell surface antibodies. An eBiosciences FOXP3 intracellular staining kit (eBioscience, 1999385) was used, and antibodies used for immune cell staining (Table S1). Data were acquired using the Thermo Fisher Attune NxT and analysed using FlowJo v10.0. Gating strategies for immune cells are shown in Figs. S1, S2. An increased proportion of cells within each tumour expressing a particular combination of immune cell-specific cell surface markers was taken as a surrogate for an increased proportion of that immune cell.

### NanoString analysis of tumours following in vivo treatment with radiotherapy

Total RNA was extracted from four 10 µm sections of formalin-fixed paraffin-embedded tissue samples using an RNeasy FFPE kit (Qiagen, 160012457). Samples were analysed using the nCounter® mouse PanCancer Immune Profiling Panel, and data acquired with the nCounter® SPRINT profiler. Data were imported into nSolver^TM^ analysis software v2.5 for quality control and normalisation of gene transcripts using NanoString analysis guidelines and housekeeping genes.

### Statistical analysis

Statistical analysis was performed using GraphPad Prism 8 (GraphPad Software, USA). For in vitro work ordinary one-way ANOVA tests were performed with Dunnett’s or Tukey’s post hoc adjustment for multiple comparisons. For in vivo tumour growth delay experiments, ordinary one-way ANOVA was performed using Tukey’s test for multiple comparisons following Brown-Forsythe’s test for equality of the means. Tumour growth delay was defined as a significant increase in time (days) for a tumour treated at 100 mm^3^ to reach end-point size of 400 mm^3^ compared against control untreated tumours, a single mouse being considered an experimental unit. *T*-tests were performed for FACS data. All results are mean ± standard error of the mean, *p* < 0.05 being statistically significant.

## Results

### MHC-I and PD-L1 expression is induced by γIFN treatment in TRAMP-C1 cells

**Fig. 1:**
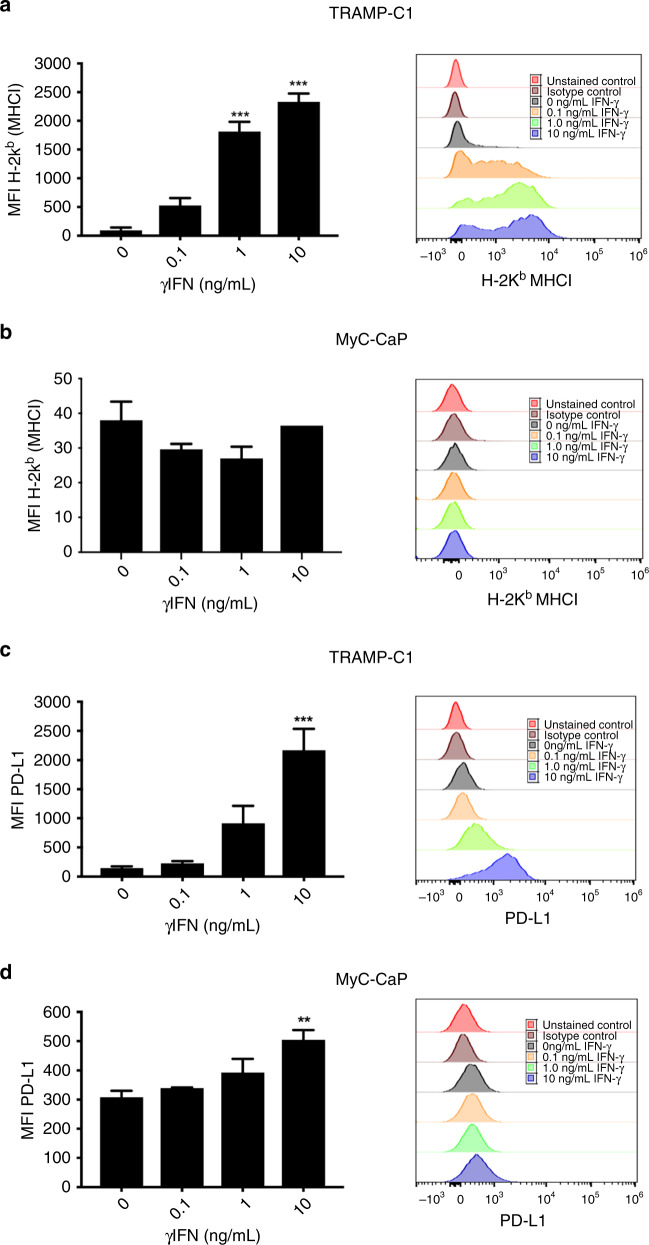
FACS analysis of MHC Class I and PD-L1 expression following in vitro treatment of TRAMP-C1 and MyC-CaP cells with γIFN. Increased expression of MHC Class I on FACS was observed in TRAMP-C1 (**a**) following 1 ng/mL and 10 ng/mL γIFN but not MyC-CaP (**b**) cells at 72 h. Increased expression of PD-L1 following in vitro treatment with 10 ng/mL γIFN was seen in both cell lines (**c**, **d**) at 72 h, with the degree of PD-L1 upregulation in response to γIFN being greater in TRAMP-C1 than in MyC-CaP cells. Data are presented as mean fluorescent intensity (MFI) ± SEM, and analysed using ordinary one-way ANOVA with Dunnett’s post hoc adjustment for multiple comparisons (*n* = 3 independent experiments). **p < 0.01; ***p < 0.001.

To select PCa cells for iRT experiments in a syngeneic immunocompetent in vivo model, TRAMP-C1 and MyC-CaP cells were chosen for in vitro and in vivo analysis. As RT induces a γIFN response cells were initially treated with γIFN, and effects on MHC-I and PD-L1 analysed with FACS. In vitro treatment of TRAMP-C1 with γIFN increased MHC-I expression in a dose-dependent manner at 72 h. This effect was not observed in MyC-CaP cells, where baseline MHC-I expression remained low with increasing γIFN (Fig. 1a, b). Increased PD-L1 expression was observed in TRAMP-C1 and MyC-CaP 72 h following 10 ng/mL γIFN, with PD-L1 upregulation following γIFN greater in TRAMP-C1 versus MyC-CaP (Fig. 1c, d). PD-L1 expression in TRAMP-C1 following 1 ng/mL γIFN peaked at 48 h and returned to baseline 120 h post-treatment (Fig. S3A). These results suggest TRAMP-C1 is a suitable model for in vivo investigation of combined anti-PD-L1 and RT.

### MHC-I and PD-L1 expression is induced by radiotherapy in TRAMP-C1 cells

**Fig. 2:**
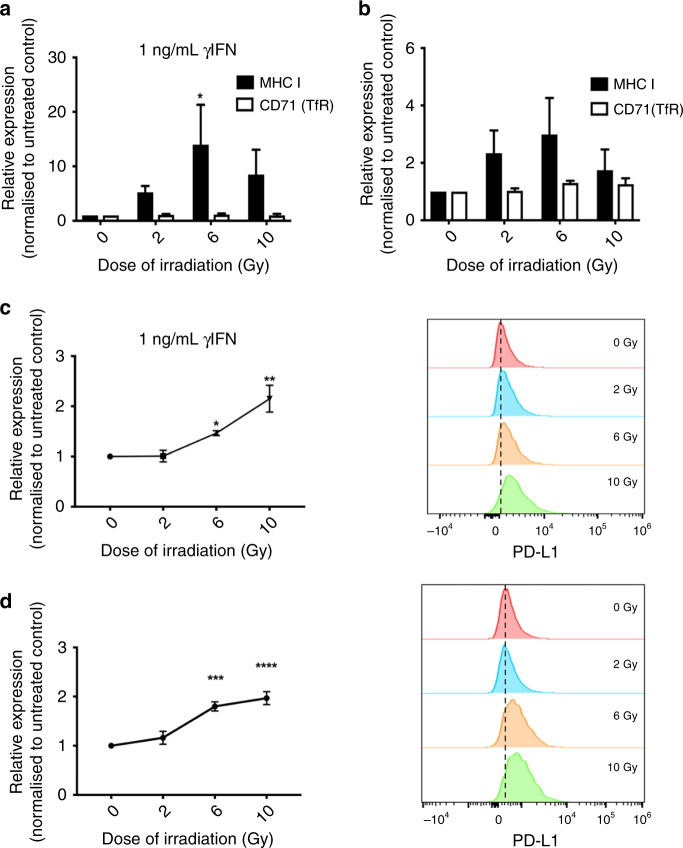
Irradiation increases prostate cancer cell surface expression of MHC Class I and PD-L1. In vitro FACS analysis of MHC Class I and control transferrin receptor expression following treatment of TRAMP-C1 cells with 6 Gy RT in the presence (**a**) or absence (**b**) of 1 ng/mL γIFN. FACS analysis of PD-L1 expression following RT in the presence (**c**) or absence (**d**) of concomitant γIFN treatment. Data are presented as relative expression normalised to untreated control and analysed using ordinary two-way ANOVA with Tukey’s post hoc adjustment for multiple comparisons (*n* = 3 independent experiments). *p < 0.05; **p < 0.01; *** *p* < 0.001, ****p < 0.0001.

To assess the suitability of TRAMP-C1 for iRT experiments combining anti-PD-L1 and RT, MHC-I and PD-L1 expression was investigated in vitro following RT. MHC-I expression was increased in TRAMP-C1 at 72 h following 6Gy RT with 1 ng/mL γIFN, whereas expression of control transferrin receptor protein remained unchanged (Fig. 2a). A non-significant increase in MHC-I expression was seen following 6 Gy without γIFN (Fig. 2b). PD-L1 expression was increased 72 h following 6 Gy and 10  Gy in the presence or absence of γIFN (Fig. 2c, d). Maximal increased PD-L1 expression in TRAMP-C1 post-6 Gy was observed 24 h later (Fig. S3B). Increased PD-L1 expression at 72 h following treatment of TRAMP-C1 with 6 Gy RT with or without 1 ng/mL γIFN was confirmed on western blot analysis (Fig. S3C). These in vitro results suggest TRAMP-C1 is a suitable PCa model for pre-clinical iRT experiments, as 6 Gy RT induces expression of MHC-I and PD-L1.

### Hypofractionated radiotherapy induces tumour growth delay in TRAMP-C1 and MyC-CaP flank prostate cancer allografts

**Fig. 3:**
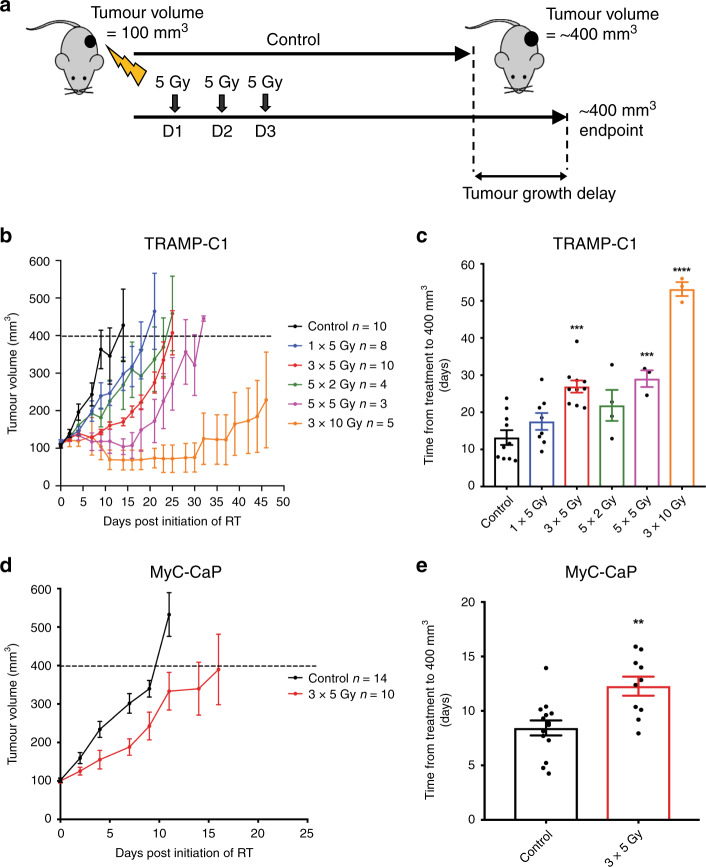
Radiotherapy causes tumour growth delay in TRAMP-C1 and MyC-CaP flank tumour allografts. Outline schematic of treatment of subcutaneous flank TRAMP-C1 and MyC-CaP tumour allografts with various RT doses and schedules (**a**). Growth kinetics of TRAMP-C1 tumours following the indicated treatments (**b**) (*n* = 10 untreated control; *n* = 8, 1 × 5 Gy RT; *n* = 10, 3 × 5 Gy RT; *n* = 4, 5 × 2 Gy; *n* = 3, 5 × 5 Gy; *n* = 5, 3 × 10 Gy). Results shown are pooled data from four experiments, with untreated control tumours included in each experiment. Median (range) body weight at treatment = 22.2 g (19.0–26.3 g). Growth kinetics of MyC-CaP tumours following the indicated treatments (**d**) (*n* = 14 untreated control; *n* = 10 3 × 5 Gy RT). Median (range) body weight at treatment = 27 g (25.3–30.4 g). Data are presented as mean tumour volume ± SEM. Tumour growth delay to ≥400 mm^3^ analysis of TRAMP-C1 (**c**) and MyC-CaP (**e**) allograft tumours treated with a range of RT doses and schedules. Data are presented as mean tumour volume ± SEM and analysed using ordinary one-way ANOVA with Tukey’s *post hoc* adjustment for multiple comparisons. *p < 0.05; **p < 0.01; *** *p* < 0.001, ****p < 0.0001.

Flank TRAMP-C1 and MyC-CaP tumour allografts were treated with 2 Gy, 5 Gy and 10 Gy RT fractions at 100 mm^3^ (Fig. 3a). Treatment of TRAMP-C1 with 3 × 5  Gy and 5 × 5 Gy resulted in significant tumour growth delay compared with untreated control tumours, however tumours eventually recurred (Fig. 3b, c). TRAMP-C1 tumour growth was well controlled with 3 × 10 Gy, however, this caused skin toxicity compared with lower doses. No significant tumour growth delay was seen with 5 × 2 Gy. Treatment of MyC-CaP with 3 × 5 Gy caused significant tumour growth delay compared with untreated control tumours, however, tumours eventually recurred (Fig. 3d, e) consistent with TRAMP-C1 results.

### Hypofractionated radiotherapy using 3 × 5 Gy induces immune changes in TRAMP-C1 and MyC-CaP flank prostate cancer allografts

**Fig. 4:**
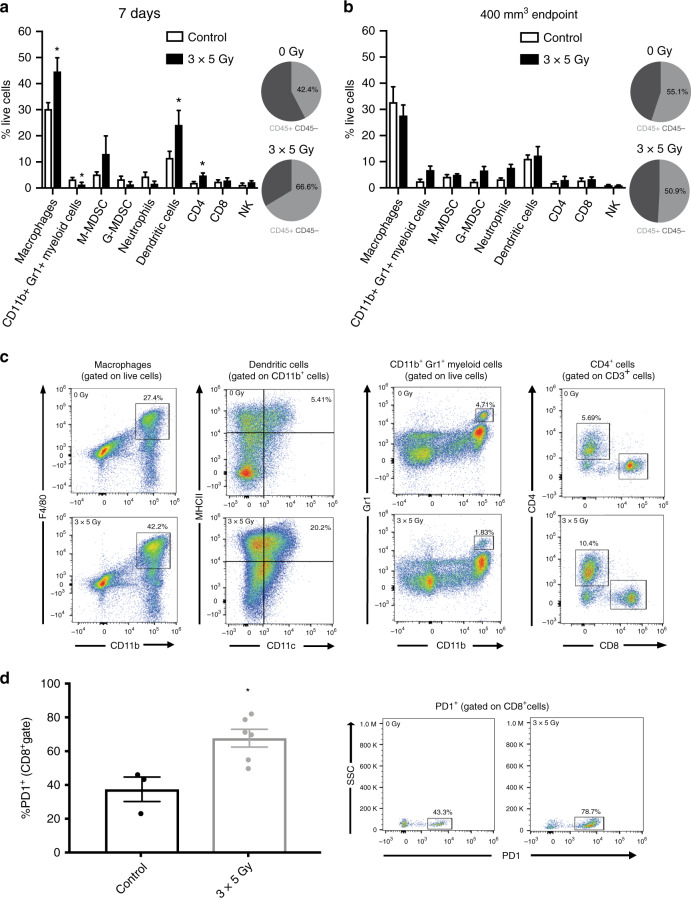
Radiotherapy causes temporal changes in the immune cell composition of TRAMP-C1 flank tumour allografts. FACS analysis of the tumour immune microenvironment of TRAMP-C1 tumour allografts at the 7-day (**a**) and tumour regrowth to ≥400 mm^3^ (**b**) time-points post initiation of 3 × 5 Gy RT compared to untreated controls, with representative plots shown (**c**). Pie charts represent the proportion of CD45^+^ leucocytes within the total population of live cells. Quantification of PD-1 expression on gated CD45^+^CD8^+^ T-cells following treatment of TRAMP-C1 allograft tumours with 3 × 5 Gy RT compared to untreated controls (**d**). Data are presented as mean percentage of total live cells ± SEM and analysed by the unpaired *t*-test (*n* = at least 4 independent tumour samples analysed) *p < 0.05.

FACS analysis of the TIME of TRAMP-C1 tumours at 7-days post initiation of 3 × 5 Gy RT demonstrated an increased proportion of cells expressing the CD45^+^ general leukocyte marker compared to control untreated tumours. The CD45^+^ immune cells were predominantly myeloid cells, with a significantly increased proportion of CD11b^+^F4/80^+^ expressing TAMs and CD11b^+^CD11c^+^MHCII^+^ expressing dendritic cells (DCs) in RT-treated tumours compared to control untreated tumours. A significantly reduced proportion of CD11b^+^Gr1^+^ expressing MDSCs was observed in 3 × 5 Gy RT-treated tumours compared to untreated controls (Fig. 4a, c). iNOS and CD206 expression (markers of M1 and M2 polarisation respectively) was analysed to define the activation status of TAMs within the TIME following 3 × 5 Gy RT. The majority of TAMs were M2-polarised in both 3 × 5 Gy RT-treated tumours and untreated control tumours, however, following 3 × 5 Gy there was a non-significant increase in iNOS-expressing TAMs (Fig. S4A, B). An increase in CD45^+^CD3^+^CD4^+^ expressing T-cells, and a non-significant increase in CD45^+^CD3^-^NK1.1^+^ expressing NK cells, was seen in 3 × 5 Gy RT-treated tumours compared to untreated control tumours. The proportion of CD45^+^CD3^+^CD8^+^ expressing T-cells and CD45^+^CD4^+^CD25^+^FoxP3^+^ expressing regulatory T-cell (Treg) cells remained unchanged in 3 × 5 Gy RT-treated tumours compared to untreated control tumours (Fig. S4C).

NanoString analysis of TRAMP-C1 tumours at 7-days post-RT revealed increased expression of CD8^+^ T-cell, DC, and Treg-specific function genes (Fig. S5). The TIME FACS and NanoString data suggest that 7-days following RT mixed immunological tumour responses have been generated. For example, the increased CD11b^+^F4/80^+^ TAMs seen on FACS may be pro-tumorigenic, whereas the increased DC number/function may have an anti-tumour effect. In contrast, at tumour regrowth post-3 × 5 Gy the TIME was similar on FACS to untreated control tumours of similar volume. There were no significant differences in the proportion of immune cell subtypes in 3 × 5 Gy RT-treated tumours versus untreated controls at tumour regrowth end-point, although there was a non-significant increase in proportion of CD11b^+^Gr1^+^ myeloid cells (*p* = 0.072), CD11b^+^Ly6C^-^Ly6G^+^ (*p* = 0.057) cells, and CD11b^+^Ly6G^+^ neutrophils (*p* = 0.070) in radio-recurrent tumours versus controls (Fig. 4b). PD-1 expression in CD45^+^CD8^+^ T-cells was upregulated in 3 × 5 Gy treated TRAMP-C1 tumours at regrowth to ≥400 mm^3^ compared to untreated control tumours (*p* = 0.02) (Fig. 4d). Corresponding NanoString analysis at tumour regrowth demonstrated that CD8^+^ T-cell, helper T-cell, and DC gene expression was reduced in radio-recurrent tumours versus controls (Fig. S6), even though the proportion of these immune populations on FACS were similar between these groups at end-point. Significantly altered NanoString immune genes at 7-day and regrowth end-points are listed for TRAMP-C1 in Tables S2, S3 and shown in Fig. S7A, B. These results illustrate that FACS and NanoString analysis of TRAMP-C1 tumours following 3 × 5 Gy suggests a DC immune response at 7-days post-treatment, along with increased PD-1 expression in CD45^+^CD8^+^ T-cells at the tumour regrowth end-point, which may be harnessed by iRT targeting PD1/PD-L1.

A similar orthogonal immune cell analysis was performed in MyC-CaP flank PCa allografts 7-days post-RT and at tumour regrowth end-point. FACS analysis of the TIME in MyC-CaP at 7-days post-RT demonstrated no change in the relative proportion of CD45^+^ cells, however a significant reduction in the proportion of CD45^+^CD3^+^CD4^+^ T-cells, and a non-significant reduction in the proportion of CD45^+^CD3^+^CD8^+^ T-cells (*p* = 0.08) was seen in treated versus untreated tumours (Fig. S8A). No significant difference in myeloid cell proportion (including TAMs, neutrophils and DCs) was observed in 3 × 5 Gy RT-treated versus control tumours, although a non-significant increase in M-MDSC infiltration (*p* = 0.075) was seen in 3 × 5 Gy RT-treated tumours. The TAM cell population in MyC-CaP was M2-polarised in 3 × 5 Gy RT-treated and untreated control tumours. A non-significant increase in iNOS-expressing TAMs following 3 × 5 Gy RT in MyC-CaP was observed (data not shown), consistent with findings in TRAMP-C1. At the tumour regrowth end-point in MyC-CaP, TIME FACS analysis demonstrated no significant differences in the proportion of myeloid immune cell subtypes in 3 × 5 Gy RT-treated versus control tumours (Fig. S8B). Analysis of the lymphoid immune cell subtypes in MyC-CaP demonstrated an increased tumour infiltrate of CD45^+^CD4^+^CD25^+^FoxP3^+^ Treg cells (Fig. S8C), and a non-significant reduction in the proportion of CD45^+^CD3^+^CD8^+^ T-cells, in 3 × 5 Gy RT-treated tumours versus untreated controls.

NanoString analysis of MyC-CaP at 7-days following 3 × 5 Gy RT showed increased heterogeneity in control tumours compared with TRAMP-C1. The analysis suggests increased expression of CD8^+^ T-cell, DC, and Treg function genes in 3 × 5 Gy RT-treated tumours compared to controls (Fig. S9), suggesting “early” anti-tumour immunogenic effects post-RT in MyC-CaP as seen in TRAMP-C1. NanoString analysis at tumour regrowth end-point in MyC-CaP demonstrated reduced DC gene expression in radio-recurrent tumours versus controls (Fig. S10).

### Concomitant inhibition of PD-L1 does not enhance anti-tumour effects of hypofractionated radiotherapy using 3 × 5 Gy in TRAMP-C1 flank prostate cancer allografts

**Fig. 5:**
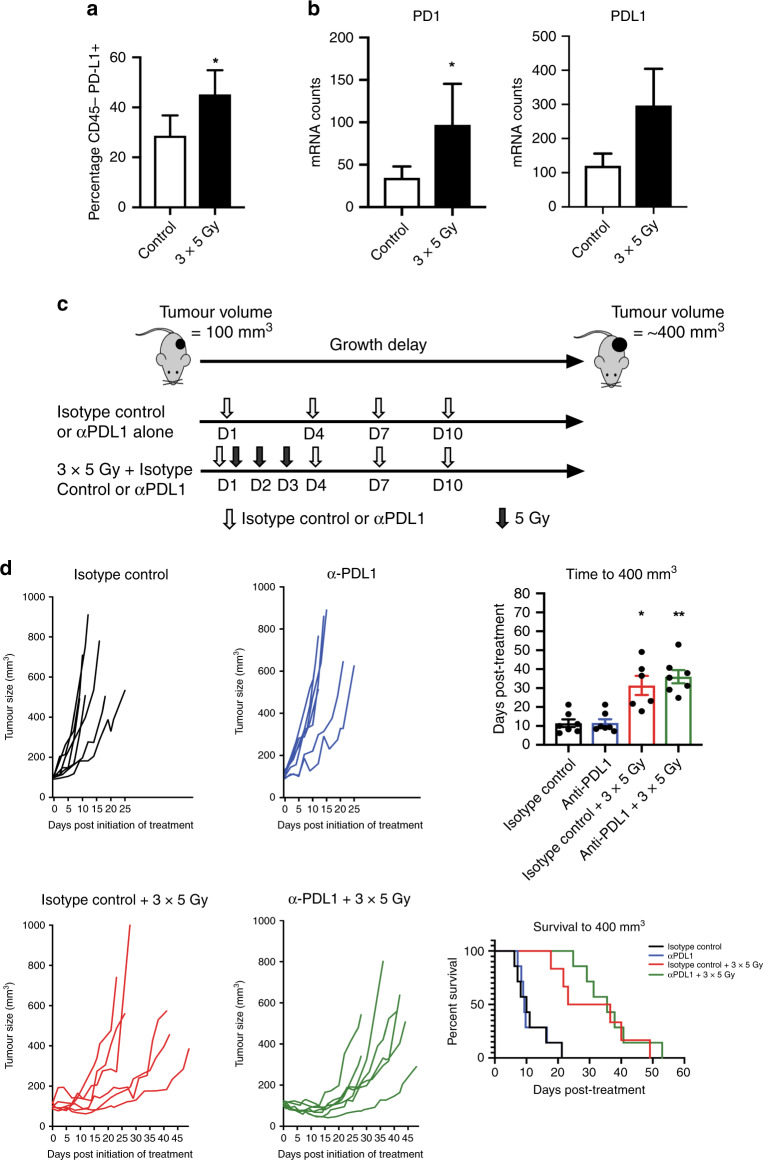
Radiotherapy upregulates the PD-1 / PD-L1 axis. Combining immunomodulation with radiotherapy does not significantly enhance tumour growth delay in TRAMP-C1 flank tumour allografts. FACS quantification of PD-L1 expression in the CD45^−^ non-immune cell population within the tumour immune microenvironment at the 4-day time-point post-initiation of 3 × 5 Gy RT compared to untreated control tumours (**a**). NanoString immune gene expression analysis of PD-1 and PD-L1 expression at the 7-day time-point post-initiation of 3 × 5 Gy RT compared to untreated control tumours (**b**). Data are presented as mean ± SEM and analysed using the unpaired *t*-test. Outline schematic of treatment of subcutaneous flank TRAMP-C1 tumour allografts with 3 × 5 Gy RT, anti-PD-L1, or a combination of 3 × 5 Gy RT and anti-PD-L1 (**c**). Tumour growth delay analysis of TRAMP-C1 allograft tumours following treatment with either 3 × 5 Gy RT alone, anti-PD-L1, or combined 3 × 5 Gy and anti-PD-L1 (**d**). The number of mice per group were: isotype control alone *n* = 7, isotype control plus RT *n* = 6, anti-PD-L1 alone *n* = 7, anti-PD-L1 plus RT *n* = 7 or isotype control. Median (range) body weight at treatment = 21.8 g (19.6–25.5 g). Data in all four treatments groups are presented as individual tumour growth kinetics, grouped tumour growth kinetics, mean ± SEM growth delay to ≥400 mm^3^, and survival to ≥400 mm^3^ using Kaplan–Meier curves. Data were analysed using ordinary one-way ANOVA with Tukey’s post hoc adjustment for multiple comparisons. *p < 0.05; **p < 0.01.

FACS analysis of the CD45^-^ non-immune cell population within the TRAMP-C1 TIME at 4-days following initiation of 3 × 5 Gy revealed increased PD-L1 expression (Fig. 5a), and NanoString analysis at 7-days demonstrated increased PD-1 expression and a non-significant increase in PD-L1 expression (Fig. 5b). To test the hypothesis that anti-PD-L1 enhances RT effects, flank TRAMP-C1 tumours were treated with 3 × 5 Gy plus anti-PD-L1, and compared against 3 × 5 Gy, anti-PD-L1, or untreated tumours (Fig. 5c). Inhibition of PD-L1, rather than combined inhibition of PD-L1 and PD-1, was chosen as iRT with 3 × 5 Gy due to concerns that combined anti-PD-L1 and anti-PD-1 might cause excessive toxicity in vivo, and given that only one agent is usually administered in the clinic. Anti-PD-L1 or isotype control was given at days 1 (along with the first 5 Gy fraction), 4, 7, and 10.^39^ Commencement of anti-PD-L1 with the first 5 Gy fraction was chosen to ensure therapeutic levels of anti-PD-L1 coincided with RT-induced PD-L1 upregulation in TRAMP-C1 tumours, and four doses were administered as this has no welfare implications in previous studies.^39^ Combined 3 × 5 Gy and anti-PD-L1 did not enhance tumour growth delay compared to 3 × 5 Gy alone in these conditions (Fig. 5d). FACS analysis of the TIME at tumour regrowth showed a non-significant increase in CD8^+^ T-cell infiltrate in tumours receiving anti-PD-L1 alone or RT plus anti-PD-L1 (Fig. S11).

## Discussion

RT is a standard treatment for localised and locally advanced PCa, and has been investigated in clinical trials treating the primary tumour in low-burden oligometastatic advanced disease.^7^ RT may cause immune-mediated anti-tumour responses^40^ through increased tumour peptide availability, tumour cell expression of MHC-I, enhanced antigen presentation, and sensitisation to tumour-specific cytotoxic lymphocytes, generation of neoantigens,^21,41–44^ and type I and type II interferon production.^45,46^ These effects sensitise irradiated tissue to tumour-specific cytotoxic lymphocytes, inducing immunological anti-tumour responses. RT may therefore “prime” the immune system to enhance anti-tumour effects of immunotherapy.^40^

Pre-clinical studies demonstrate that tumour cell MHC-I expression is a surrogate marker of immunogenicity, and correlates with increased cytotoxic T-cells in the TIME and increased response of tumour models to immunotherapy, with an inverse relation to tumour growth rate.^47,48^ We investigated baseline in vitro cell surface expression of MHC-I and PD-L1 in TRAMP-C1 and MyC-CaP cells, along with the response to γIFN, as a measure of de novo immunogenicity. These results suggest TRAMP-C1 has a greater potential for immunogenicity than MyC-CaP, given the higher MHC-I and PD-L1 expression at baseline and following γIFN. This is supported by observations in TRAMP-C1 allografts versus MyC-CaP; a greater CD45^+^ cell infiltrate at baseline and following RT; RT-induced altered expression of immune-related genes on NanoString; and reduced tumour growth rate. Increased cell surface MHC-I expression following RT may be due to direct effects on the pool of poly-ubiquitinated proteins targeted for proteosomal degradation, resulting in more peptides for MHC-I antigen presentation, and enhanced MHC-I expression.^49^ Consistent with the previous reports^21,50,51^ we observed RT increased MHC-I expression in TRAMP-C1 tumour allografts. These observations suggest TRAMP-C1 is relatively immunogenic and a suitable pre-clinical model to investigate iRT.

RT has been regarded as immunosuppressive and potentially inappropriate for combination with immunomodulation.^52^ However, RT may also be an immunostimulatory anti-PCa therapy,^52^ and the relationship between RT and immunoreactivity is complex. Data on use of RT and immunotherapy are accumulating and suggest opportunities for RT to “prime” the immune system and enhance anti-tumour effects of immunotherapy.^46^ FACS analysis of the TRAMP-C1 TIME following 5 Gy fractions suggests an “early” predominantly immunostimulatory TIME 7-days post-RT, with increased DCs and CD4^+^ T-helper cells, and reduced MDSCs. NanoString analysis in TRAMP-C1 allografts at this 7-day time-point suggests anti-tumour immunogenic effects, with increased expression of CD8^+^ T-cell and DC function genes. The lack of increased infiltration of cytotoxic CD8^+^ T-cells in the TIME on FACS may be due to use of 5 Gy RT fractions, and higher fraction sizes doses may be necessary to achieve this effect. Despite 3 × 5 Gy RT achieving a significant tumour growth delay and predominantly immunostimulatory effects, treatment was sub-lethal and failed to generate a sufficient anti-tumour response for cure. In contrast, and supporting potential immunosuppressive effects of RT, “early” time-point FACS in MyC-CaP demonstrated significantly reduced CD4^+^ helper T-cells, increased CD4^+^ Treg cells, and a trend towards a reduction in the proportion of the CD8^+^ T-cell population.

Pre-clinical PCa models, such as TRAMP-C2 allografts, have demonstrated barriers to generating anti-tumour immune responses, including poor CD8^+^ T-cell infiltrates, and high intra-tumoural proportions of immunosuppressive myeloid cells such as TAMs and MDSCs.^53^ We observed poor infiltration of CD8^+^ T-cells in TRAMP-C1, with TAMs and MDSCs comprising the majority of CD45^+^ cells. Whilst RT doses in the literature are higher than the 3 × 5 Gy used in our experiments, our observation of an increase in CD11b^+^F4/80^+^ expressing TAMs 7-days post-3 × 5 Gy is consistent with observations in TRAMP-C1.^14^ TAMs are myeloid cells present in many solid-organ tumours including PCa, and they demonstrate functional plasticity, differentiating into various phenotypes during inflammatory responses. TAMs are described as “M1” with predominantly anti-tumorigenic effects, or “M2” with predominantly pro-tumorigenic effects such as angiogenesis and immunosuppression. Our analysis of TAM activation status using iNOS and CD206 suggests M2 TAMs are predominantly present in the TRAMP-C1 TIME. In TRAMP-C1 allografts treatment with 3 × 5 Gy recruited both M1 and M2 TAMs, and we speculate that blocking this recruitment may enhance anti-tumour RT responses. The observed trend towards an increased proportion of MDSCs at the “late” tumour regrowth time-point following 3 × 5 Gy RT may result in immunosuppression and CD8^+^ T-cell exhaustion, beyond that induced by a RT-induced influx of TAMs. The observation that PD-1 expression is increased in CD8^+^ T-cells at tumour regrowth following 3 × 5 Gy RT suggests the presence of T-cell exhaustion in TRAMP-C1, and the reduced CD8^+^ T-cell, helper T-cell, and DC gene expression on NanoString analysis in radio-recurrent compared with control untreated tumours suggests an immunosuppressed TIME at eventual tumour re-growth. Further experiments to target this effect are warranted.

Differentially expressed genes in TRAMP-C1 at the “early” 7-day time-point post-RT included upregulation of chemokine genes such as CCL2 and its receptor CCR2, CCL17, CCL22, CXCR3 and CCL7. Chemokines and their receptors are expressed by cancer cells and play roles in tumour progression and therapeutic outcomes. Several chemokines and their receptors, in particular CCL2 and CCR2, are implicated in PCa progression, metastasis and chemoresistance,^54,55^ and may be expressed by tumour cells or TAMs recruited to the TIME. Tumour-derived CCL2 mediates RT resistance in pre-clinical pancreatic ductal adenocarcinoma through recruitment of TAMs and MDSCs, supporting cancer cell proliferation and neovascularisation post-RT.^56^ Approaches to mitigate immunosuppressive TIME effects of RT-induced TAMs by blocking CCL2-CCR2, or depleting these cells using concomitant anti-CSF with RT, may be necessary in PCa to derive benefit from iRT.^57^

Analysis of TRAMP-C1 tumours demonstrated that the PD-1/PD-L1 axis is upregulated in the TIME following 3 × 5 Gy. We tested the hypothesis that iRT through PD-L1 inhibition with concomitant 3 × 5 Gy RT may enhance tumour growth delay compared to RT alone. There are several possible explanations for the absence of a demonstrable increase in tumour growth delay through iRT in these conditions in this model. The 3 × 5 Gy RT dose may be insufficient to generate CD8^+^ T-cell dependent anti-tumour responses that can be further harnessed through anti-PD-L1. Moreover, combined anti-PD-1 and anti-PD-L1 with RT may be necessary to harness a response through enhanced blockade of the PD-1/PD-L1 axis, however, we did not test this combination due to concerns about possible in vivo toxicity, and given that combined anti-PD-1 and anti-PD-L1 is not standard in the clinic. The tumour growth delay and survival curve analysis suggest a possible initial enhanced response to anti-PD-L1 with RT compared to RT alone, however, this is not maintained, with no difference once the anti-PD-L1 is no longer administered. It may be the case that administration of anti-PD-L1 for a longer time period than the x4 doses delivered over 12 days in our model and as previously described^39^ may be necessary. It is possible that the anti-PD-L1 needs to be continued for several weeks post-RT, however, we did not pursue this approach due to concerns that prolonged anti-PD-L1 may be poorly tolerated and lead to significant toxicity. It is also possible that the experiment was under-powered given the variable response of TRAMP-C1 tumours to 3 × 5 Gy RT, with three of the combined treatment animals demonstrating enhanced response, whilst a further three did not, leading to no significant difference. Future experiments using a longer time course of concomitant anti-PD-L1, and using combined anti-PD-1 and anti-PD-L1 and RT, subject to acceptable toxicity in vivo, may help to answer these issues.

Previous experiments in autochthonous TRAMP models developing spontaneous PCa suggest that neither RT (administered as 3 × 10 Gy, with a 5-day interval between doses) nor immunotherapy alone (in the form of a tumour vaccine) can prime an anti-tumour immune response in animals with evolving tumours, but that iRT could result in anti-tumour T cell activation, although this effect was dependent on the relative timing of RT and immunotherapy.^10^ Anti-tumour immune responses occurred when immunotherapy was administered 3–5 weeks post-RT, but these responses were undetectable when immunotherapy was administered at either earlier (peri-RT) or later time-points. It is possible that there is a relatively narrow therapeutic temporal window of opportunity for immunotherapy post-RT to be effective in highly aggressive immunosuppressive tumour models such as the autochthonous TRAMP model and our TRAMP-C1 flank allograft models, albeit these studies used different RT doses and fractionation. The TIME results from our experiments suggest that despite RT, the CD8^+^ T-cells which play important roles in the anti-tumour effects of ablative RT^58^ remain at low levels following 3 × 5 Gy RT, despite iRT with anti-PD-L1. This observation suggests that therapeutic strategies combining RT with T-cell-based therapies such as immune checkpoint blockade may not be clinically effective without first finding strategies to enhance T-cell immunosurveillance. Monocytes, in particular, play important roles in the immune evasion/tolerance of PCa,^59^ and it may be that immunotherapy directed at inhibiting TAMs or MDSCs recruitment to the TIME in combination with RT are needed to improve disease control in PCa.

These experiments have several limitations. Flank tumour allografts were used, rather than orthotopic tumours, or de novo PCa arising within transgenic mice, which might better recapitulate the TIME of spontaneously arising PCa. Standard implanted tumour models may not be ideal for iRT studies as they may fail to properly model the physiological setting in which tolerogenic tumours arise over extended time periods. However, the flank allograft tumour approach within immunocompetent syngeneic mice was necessary to deliver iRT with follow-up and measurement of tumour growth. It is possible that the length of time between subcutaneous injection of TRAMP-C1 cells and formation of 100 mm^3^ tumours, which we found to be 6–8 weeks in the conditions tested, led to changes in the immunogenicity of these tumours, perhaps with the development of immune tolerance in the animals, such that iRT was less effective. A variation in the efficacy of immunomodulation, dependent upon the timing of initiation of treatment following tumour implantation, and consistent with a decay of the initial immune response following tumour implantation has been reported.^60^ However, failure of delayed immunomodulation may be due to accumulation of a greater tumour burden or acquisition of an immunosuppressed TIME. RT may need to be continued for a greater number of fractions than 3 × 5 Gy to enhance effects of anti-PD-L1. This warrants further analysis, provided animals tolerate a higher number of RT fractions over several weeks, and given that RT is administered under general anaesthetic. An additional limitation of these experiments is the absence of concomitant ADT, given that this is administered with 2 Gy RT fractions as standard-of-care in patients. However, adding ADT would have introduced a third variable (in addition to RT and anti-PD-L1), and the TRAMP-C1 model is androgen independent. Moreover, recent clinical trials such as PACE (Prostate Advances in Comparative Evidence)^15^ are investigating hypofractionated RT without ADT versus conventional RT plus ADT. It may be the case that ADT plus RT leads to enhanced anti-tumour responses through addition of anti-PD-L1, and this possibility warrants investigation in future studies.

The possibility for an iRT approach to be beneficial in patients with PCa is an important idea to test given that the 5-year biochemical relapse rate for high-risk patients is 32–70%.^3^ If iRT was successful then this may permit a reduction in the necessary RT dose for effective treatment, and could have clinical benefit for patients as RT reduces quality of life due to side effects.^4–6^ Immunotherapy is an attractive option combined with RT given recent clinical trial results where RT to the primary tumour in oligometastatic disease led to clinical benefit,^7^ suggesting possible RT-induced immunological responses which may be enhanced with iRT. However, for iRT to be successfully used in the clinic, studies need to be performed in pre-clinical models and early phase clinical trials to determine the optimal timing, sequence of delivery, and type of immunomodulation used in iRT.

In conclusion, 3 × 5 Gy hypofractionated RT resulted in a tumour growth delay in MyC-CaP and TRAMP-C1 PCa models. 3 × 5 Gy resulted in an initial increase in TAMs and DCs, and upregulation of PD-1/PD-L1, in the TRAMP-C1 TIME, however, anti-PD-L1 combined with RT in the conditions tested did not increase tumour growth delay. These results suggest that adjuncts beyond immunomodulation, such as perhaps combination with PCa focal ablation therapies or other cancer cell-targeting treatments, which warrants evaluation in future studies, are necessary to improve the anti-tumour response from RT fraction sizes used in the clinic.

## Supplementary information

Supplementary figure legends

Figure S1

Figure S2

Figure S3

Figure S4

Figure S5

Figure S6

Figure S7

Figure S8

Figure S9

Figure S10

Figure S11

Table S1

Table S2

Table S3

## Data Availability

The authors agree to make the data in this paper publically available.
