# Supplementary material for: Impacts of combining anti-PD-L1 immunotherapy and radiotherapy on the tumour immune microenvironment in a murine prostate cancer model
Source: Br J Cancer. 2020 Jul 9;123(7):1089–100. doi: 10.1038/s41416-020-0956-x (PMC7525450; doi:10.1038/s41416-020-0956-x)
Supplement: Supplementary file 1 — Supplementary figure legends [file 41416_2020_956_MOESM1_ESM.docx]

**Supplementary figure legends**

**Figure S1.**

Representative flow cytometry dot plots indicating the gating strategy for identification of myeloid populations. Gates were used to exclude debris (**A**), doublets (**B**) and dead cells (**C**). Live cells were gated for subsequent analysis of macrophages (**E**), MDSCs (**F**) and neutrophils (**G**). CD11b^+^ cells (**D**) were gated for subsequent analysis of Ly6C^+^ (monocytic MDSCs) versus Ly6G^+^ (granulocytic MDSCs) (**J**) and CD11c^+^ and MHCII^+^ expression (**K**). CD11b^+^F4/80^+^ macrophages (**E**) were gated, and iNOS^+^(**H**) and CD206^+^ (**I**) expression measured. iNOS^+^ and CD206^+^ isotype controls and fluorescence minus one (FMO) controls for Ly6G^+^, Ly6C^+^, MHCII^+^ and CD11c^+^ are shown (**L-Q**).

**Figure S2.**

Representative flow cytometry dot plots indicating the gating strategy for identification of lymphoid and Treg populations. Gates were used to exclude debris (**A**), doublets (**B**) and dead cells (**C**). CD45^+^ cells (**D**) were subsequently analysed for NK1.1^+^ (**E**), CD3^+^ (**F**), and CD4^+^ or CD8^+^ (**G**). CD45^+^CD3^+^CD8^+^ cells were gated for PD1^+^ expression (**I**). FMO for NK1.1 is shown in panel **H**. For Treg cells, CD45^+^ cells (**M**) were gated for CD4^+^ expression followed by CD25^+^ and FoxP3^+^ expression (**O**). FMO controls for CD25^+^ and FoxP3^+^ are shown (**P**, **Q**).

**Figure S3**

Time course FACS quantification of PD-L1 expression on TRAMP-C1 cells following 1ng/mL γIFN (**A**) and 6Gy RT (**B**). Western blot analysis of TRAMP-C1 cell lysates following *in vitro* treatment with 6Gy RT in the presence of 1ng/mL γIFN confirmed increased expression of PD-L1 at 72 hours (**C**). The Western blot is a representative image from each of three independent experiments, each performed in duplicate.

**Figure S4**

FACS quantification analysis of iNOS (**A**) and CD206 (**B**) expression on gated macrophages (CD11b^+^ F4/80^+^) from TRAMP-C1 tumours with representative histograms following 3x5Gy RT compared to control untreated tumours. Quantification of CD45^+^CD4^+^CD25^+^FoxP3^+^ regulatory T-cell cells following 3x5Gy RT compared to control untreated tumours (**C**). Data is presented as mean ± SEM, and analysed using the unpaired t-test.

**Figure S5**

Heatmaps of changes in RNA expression of genes associated with immune cell subsets in TRAMP-C1 using NanoString following 3x5Gy RT compared to untreated controls. RNA was extracted from tumours on day 7 following initiation of RT.

**Figure S6**

Heatmaps of changes in RNA expression of genes associated with immune cell subsets in TRAMP-C1 using NanoString following 3x5Gy RT compared to untreated controls. RNA was extracted from tumours when tumour volume reached ≥400mm^3^ following initiation of RT.

**Figure S7**

Volcano plots demonstrating Log2 (fold change) of genes in the “early” 7-day (**A**) and “late” tumour regrowth to ≥400mm^3^ (**B**) time points for TRAMP-C1 tumours treated with 3x5Gy RT compared to untreated controls. Significantly (*p*<0.05) up-regulated or down-regulated genes are annotated on the volcano plot

**Figure S8**

FACS analysis of the tumour immune microenvironment of MyC-CaP tumour allografts at the 7-day (**A**) and tumour regrowth to ≥400mm^3^ (**B**) time-points post initiation of 3x5Gy RT compared to non-irradiated controls, with representative plots (**D**). Pie charts represent the proportion of CD45^+^ leucocytes within the total population of live cells. Quantification of CD45^+^CD4^+^CD25^+^FoxP3^+^ regulatory T-cell cells following 3x5Gy RT compared to control untreated tumours (**C**). Data are presented as mean percentage of total live cells ± SEM, and analysed using the unpaired t-test (n = at least 5 independent tumour samples analysed). **p*<0.05.

**Figure S9**

Heatmaps of changes in RNA expression of genes associated with immune cell subsets in MyC-CaP using NanoString following 3x5Gy RT compared to untreated controls. RNA was extracted from tumours on day 7 following initiation of 3x5Gy RT.

**Figure S10**

Heatmaps of changes in RNA expression of genes associated with immune cell subsets in MyC-CaP using NanoString following 3x5Gy RT compared to untreated controls. RNA was extracted from tumours when tumour volume reached ≥400mm^3^ following initiation of RT.

**Figure S11**

FACS analysis of the tumour immune microenvironment at eventual tumour regrowth to ≥400mm^3^ in TRAMP-C1 allograft tumours following treatment with either 3x5Gy RT, anti-PD-L1 alone, or combined 3x5Gy and anti-PD-L1.
