# Supplementary figures and images for: Impacts of combining anti-PD-L1 immunotherapy and radiotherapy on the tumour immune microenvironment in a murine prostate cancer model
Source: Br J Cancer. 2020 Jul 9;123(7):1089–100. doi: 10.1038/s41416-020-0956-x (PMC7525450; doi:10.1038/s41416-020-0956-x)

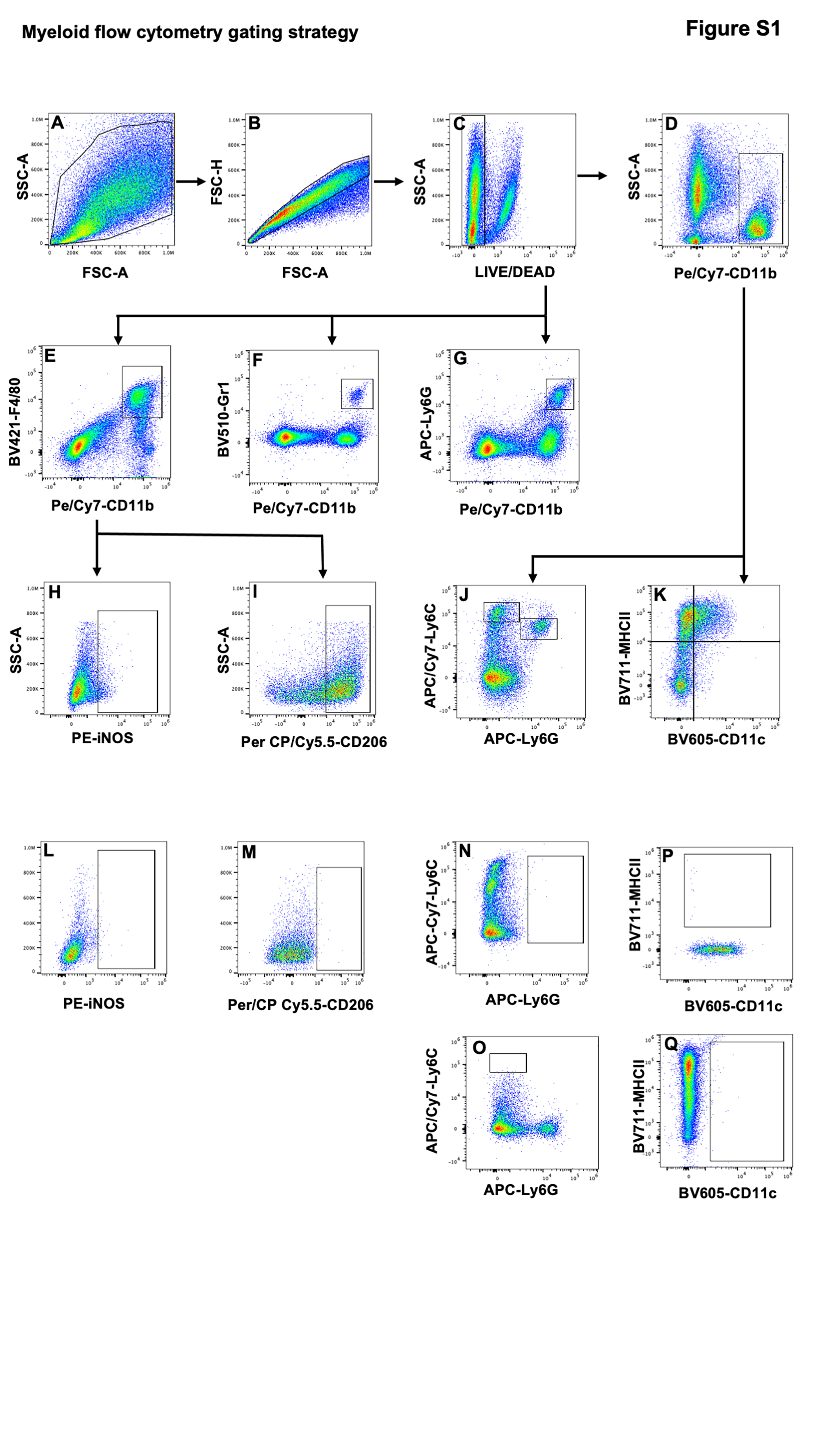

Supplement: Supplementary file 2 — Figure S1 [file 41416_2020_956_MOESM2_ESM.tif]

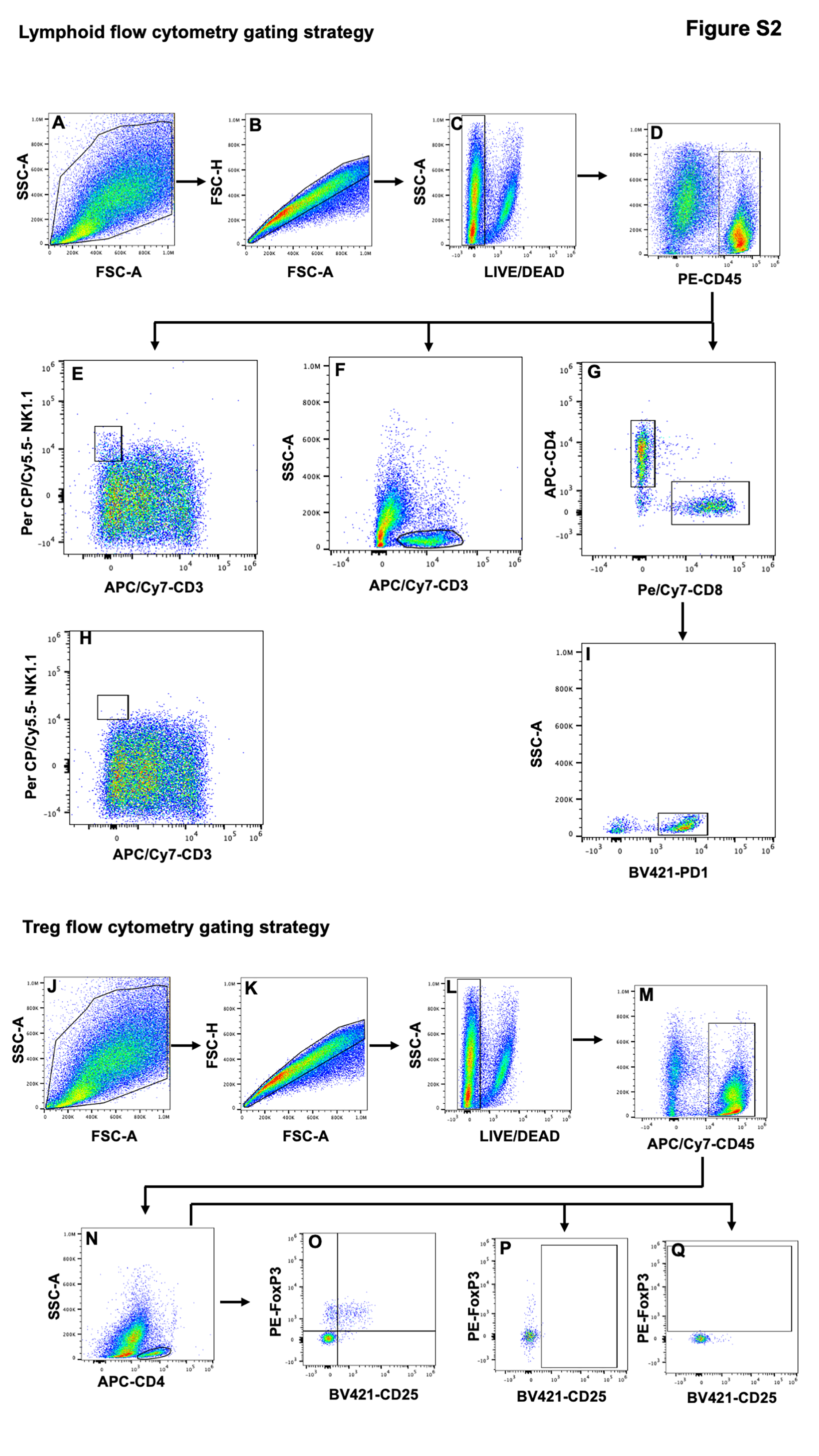

Supplement: Supplementary file 3 — Figure S2 [file 41416_2020_956_MOESM3_ESM.tif]

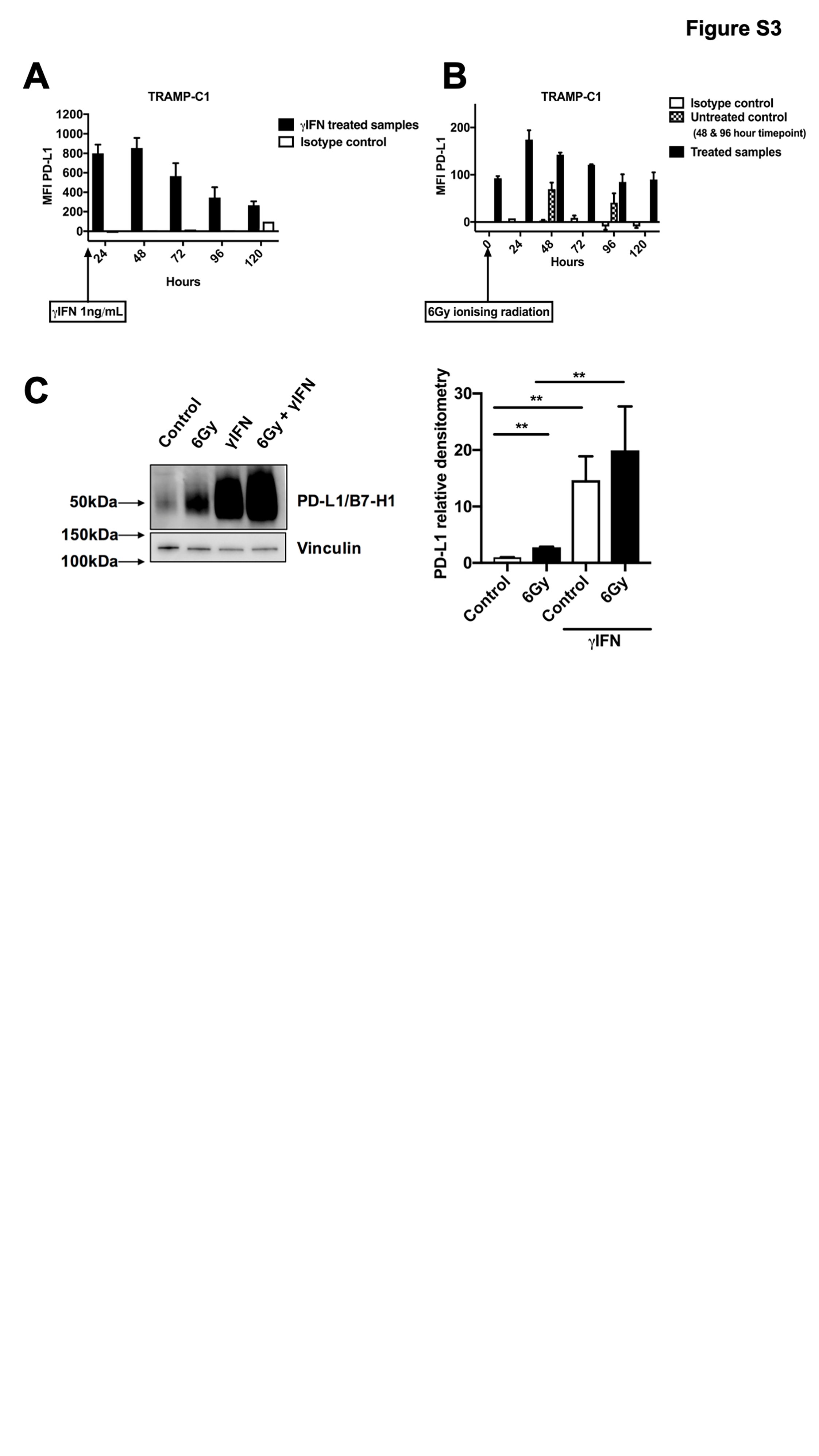

Supplement: Supplementary file 4 — Figure S3 [file 41416_2020_956_MOESM4_ESM.tif]

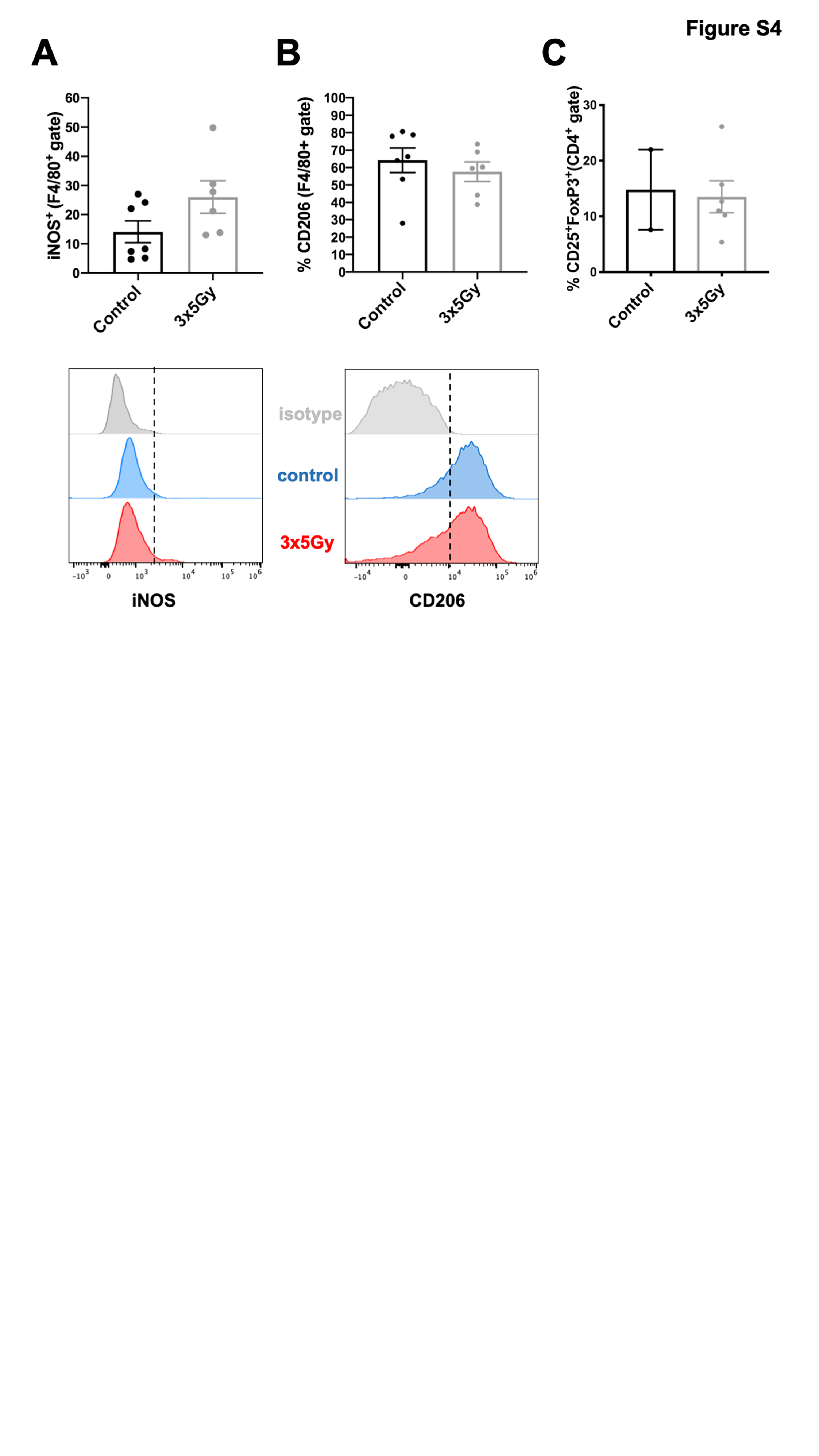

Supplement: Supplementary file 5 — Figure S4 [file 41416_2020_956_MOESM5_ESM.tif]

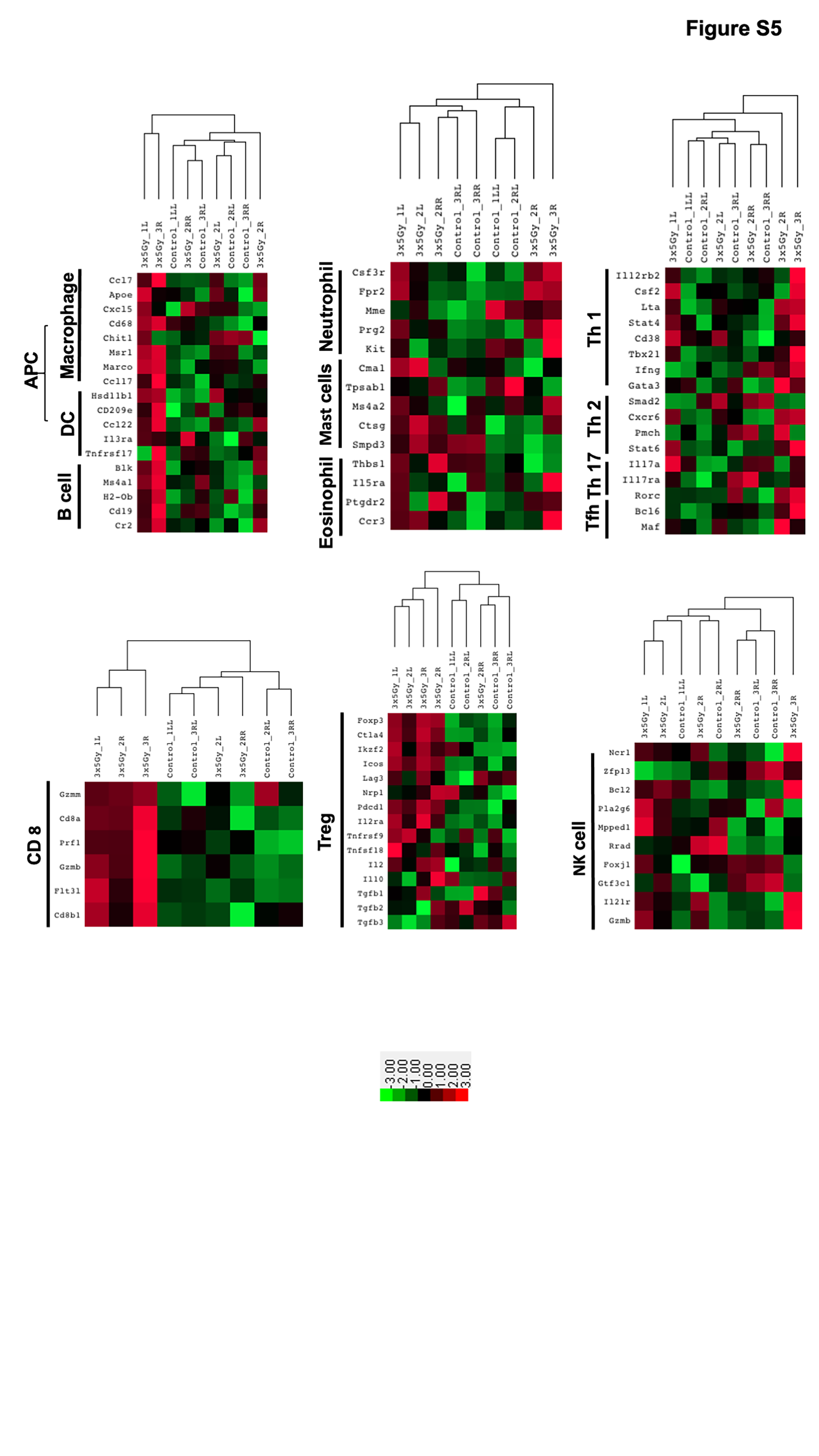

Supplement: Supplementary file 6 — Figure S5 [file 41416_2020_956_MOESM6_ESM.tif]

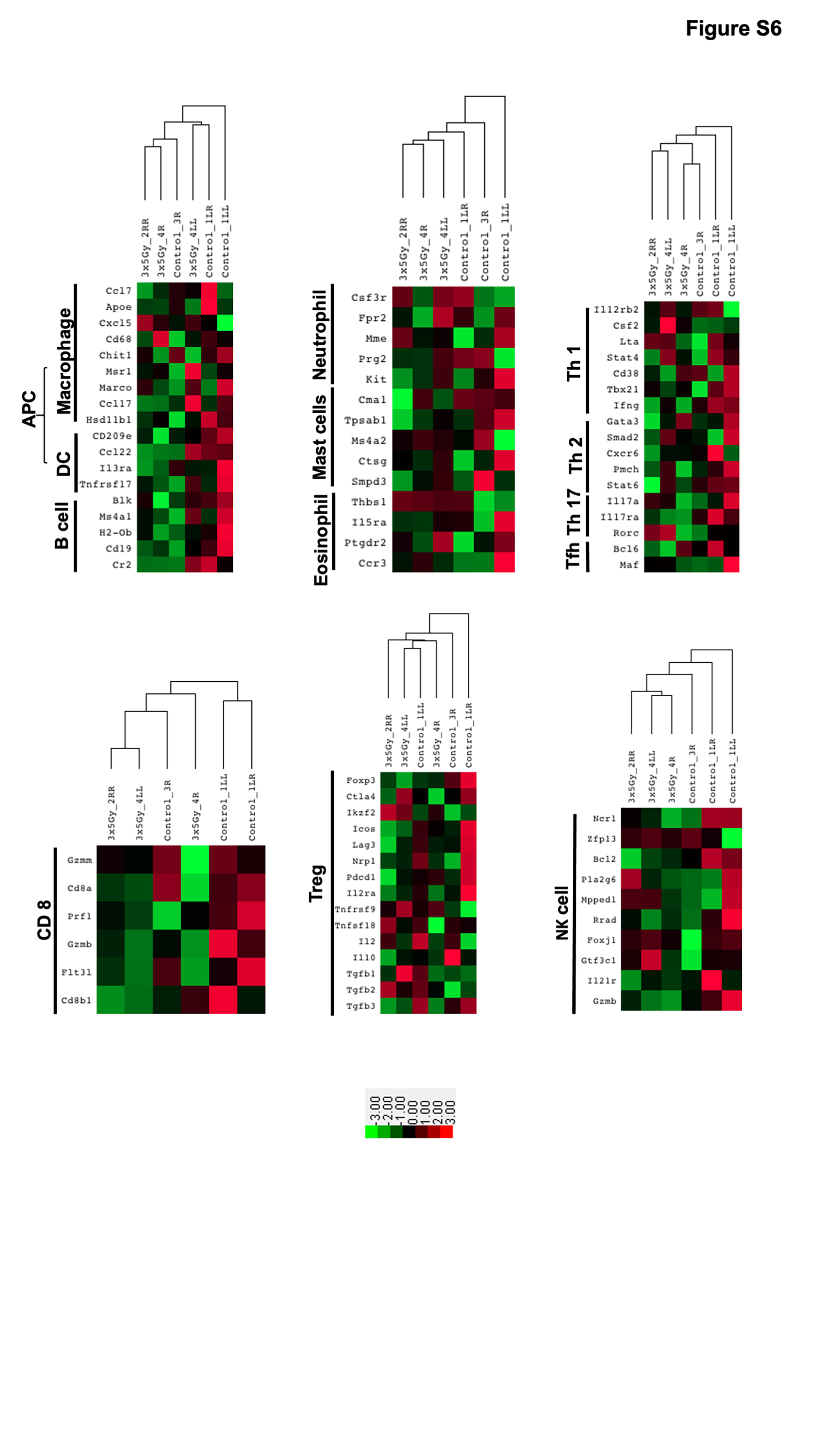

Supplement: Supplementary file 7 — Figure S6 [file 41416_2020_956_MOESM7_ESM.tif]

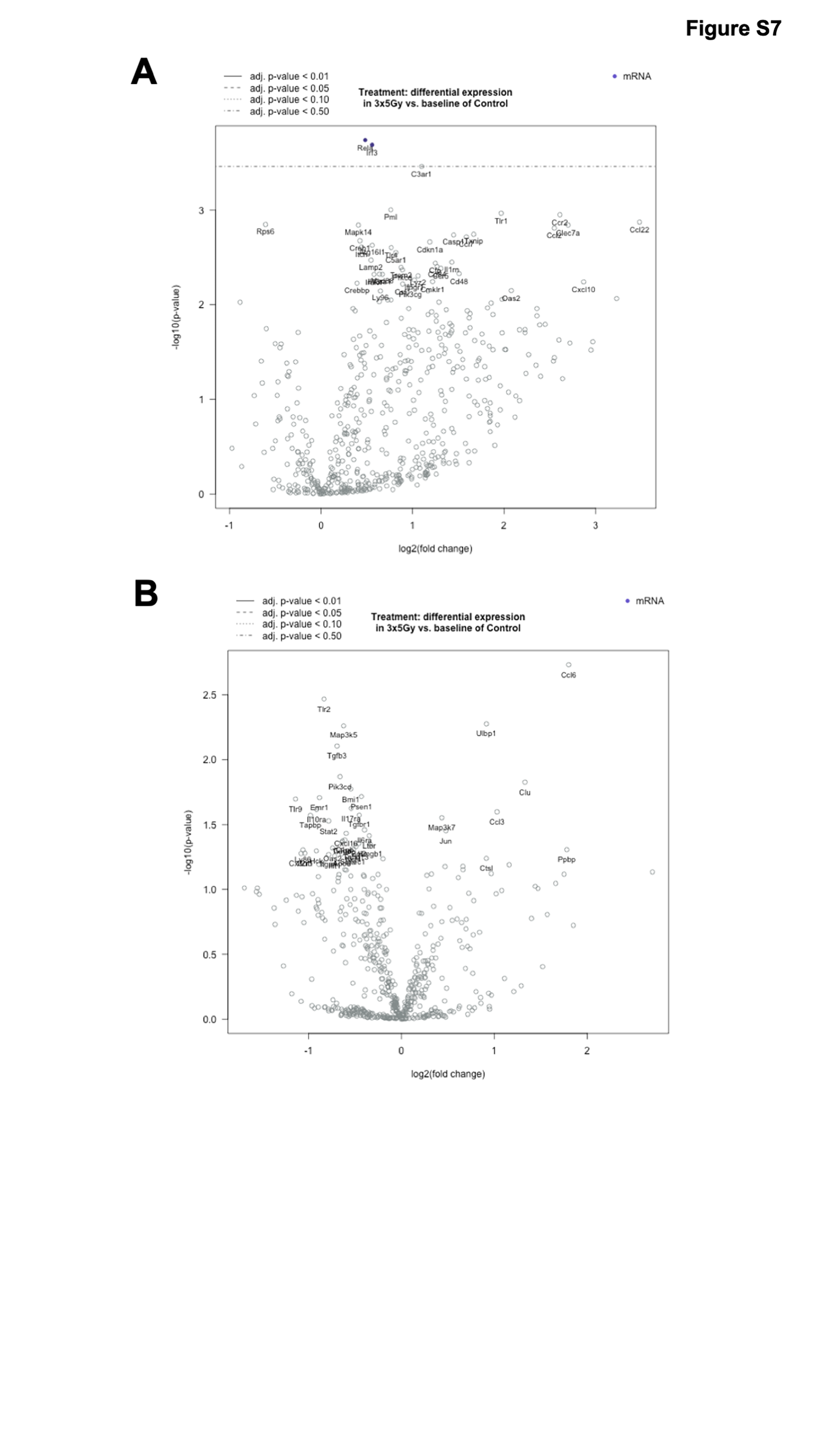

Supplement: Supplementary file 8 — Figure S7 [file 41416_2020_956_MOESM8_ESM.tif]

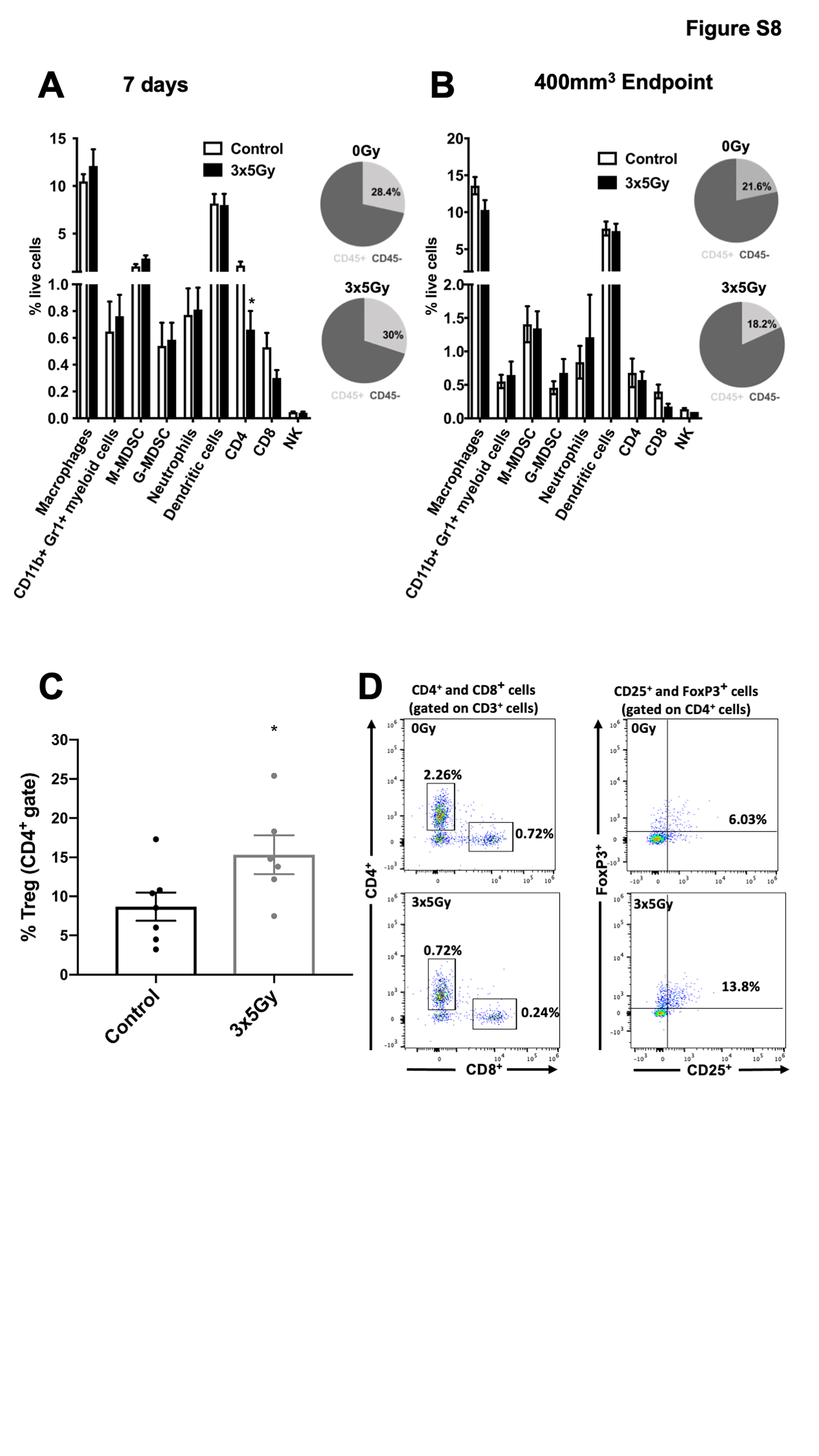

Supplement: Supplementary file 9 — Figure S8 [file 41416_2020_956_MOESM9_ESM.tif]

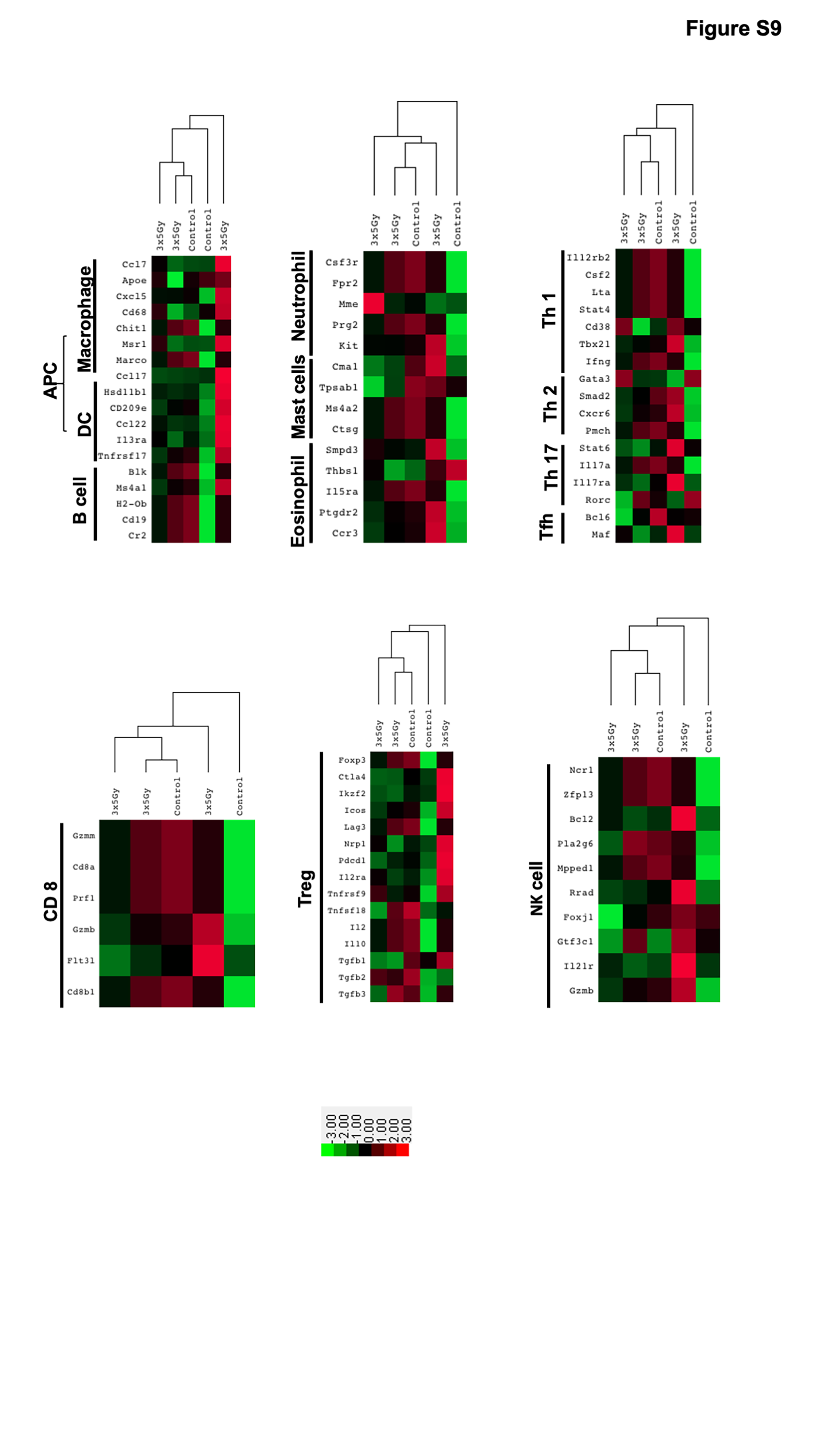

Supplement: Supplementary file 10 — Figure S9 [file 41416_2020_956_MOESM10_ESM.tif]

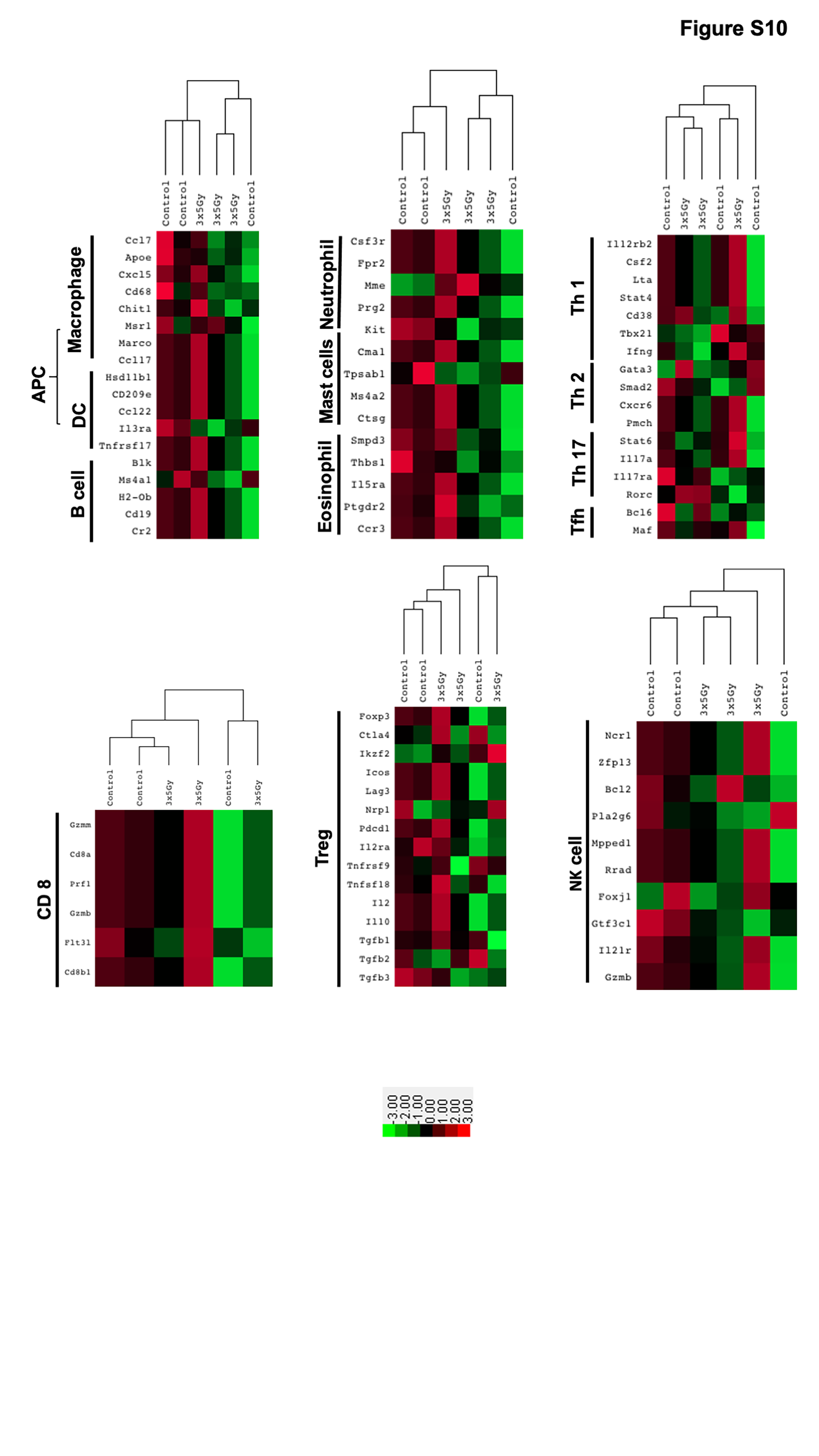

Supplement: Supplementary file 11 — Figure S10 [file 41416_2020_956_MOESM11_ESM.tif]

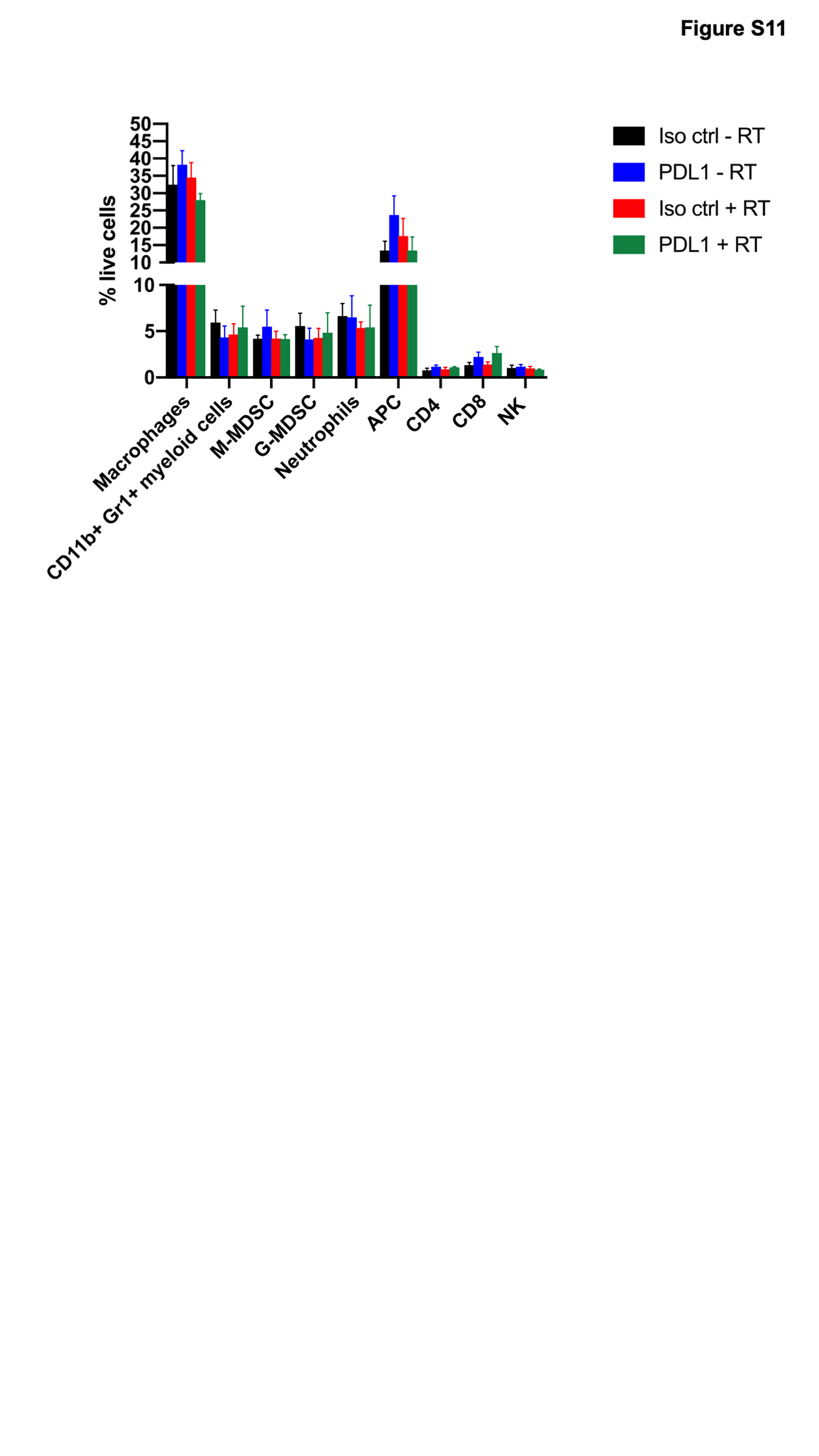

Supplement: Supplementary file 12 — Figure S11 [file 41416_2020_956_MOESM12_ESM.tif]
