# Supplementary material for: Impacts of combining anti-PD-L1 immunotherapy and radiotherapy on the tumour immune microenvironment in a murine prostate cancer model
Source: Br J Cancer. 2020 Jul 9;123(7):1089–100. doi: 10.1038/s41416-020-0956-x (PMC7525450; doi:10.1038/s41416-020-0956-x)
Supplement: Supplementary file 13 — Table S1 [file 41416_2020_956_MOESM13_ESM.docx]

| **Panel** | **Antibodies** |
| --- | --- |
| Tumour infiltrating myeloid cells | PE-Cy7 CD11b (eBioscience – M1/70), PerCP/Cy5.5 CD206 (BioLegend – C068C2), BV510 Gr-1 (BioLegend – RB6-8C5), BV421 F4/80 (BioLegend – BM8), APC Ly6G (BioLegend – 1A8), APC-Cy7 Ly6C, (BioLegend – HK1.4), BV605 CD11c (BioLegend – N418), BV711 I-A/I-E (BioLegend – M5/114.15.2), PE iNOS2 (Invitrogen – CXNFT), LIVE/DEAD™ Fixable Green Dead Cell Stain Kit (ThermoFisher Scientific - L23101) |
| Tumour infiltrating lymphoid cells | PE CD45 (invitrogen – 30-F11), APC-Cy7 CD3 (BioLegend – 17A2), APC CD4 (BioLegend – RM4-5), PECy7 CD8 (BioLegend – 53-6.7), PerCP/Cy5.5 NK1.1 (BioLegend - PK136), LIVE/DEAD™ Fixable Green Dead Cell Stain Kit (ThermoFisher Scientific - L23101) |
| CD8^+^/CD4^+^ T-cell PD1 | PE CD45 (Invitrogen – 30-F11), CD8a BioLegend – 53-6.7), APC CD4 (BioLegend – RM4-5), BV421 PD1 (BioLegend – 29F.1A12), LIVE/DEAD™ Fixable Green Dead Cell Stain Kit (ThermoFisher Scientific - L23101) |
| CD4^+^ regulatory T-cells | APC-Cy7 CD45 (BioLegend – 30-F11), APC CD4 (BioLegend – RM4-5), BV421 CD25 (BioLegend 29F.1A12), PE Anti-Mo FoxP3 (invitrogen – NRRF–30), LIVE/DEAD™ Fixable Green Dead Cell Stain Kit (ThermoFisher Scientific - L23101) |

**Table S1.**

Antibodies and markers used in flow cytometry to discriminate different immune cell subtypes.
