# Supplementary material for: Impacts of combining anti-PD-L1 immunotherapy and radiotherapy on the tumour immune microenvironment in a murine prostate cancer model
Source: Br J Cancer. 2020 Jul 9;123(7):1089–100. doi: 10.1038/s41416-020-0956-x (PMC7525450; doi:10.1038/s41416-020-0956-x)
Supplement: Supplementary file 14 — Table S2 [file 41416_2020_956_MOESM14_ESM.docx]

| **Gene** | ***p*-value** |
| --- | --- |
| C3ar1 | 0.000218 |
| Rela | 0.00022 |
| Irf3 | 0.00101 |
| Cdkn1a | 0.00159 |
| Itch | 0.00226 |
| Rps6 | 0.00234 |
| Trem2 | 0.00239 |
| Clec7a | 0.00242 |
| C5ar1 | 0.00275 |
| Tnfsf13 | 0.00291 |
| Pml | 0.00295 |
| Tlr1 | 0.00392 |
| Ccr2 | 0.00402 |
| Bmi1 | 0.00473 |
| Ccl2 | 0.00486 |
| Mapk14 | 0.00489 |
| Tlr4 | 0.00547 |
| Ccl7 | 0.00548 |
| Atm | 0.00555 |
| Psmd7 | 0.00573 |

**Table S2**

Significantly altered NanoString immune genes at the “early” 7-day time point following 3x5Gy radiotherapy treatment of TRAMP-C1 flank tumour allografts.
