# Supplementary material for: Impacts of combining anti-PD-L1 immunotherapy and radiotherapy on the tumour immune microenvironment in a murine prostate cancer model
Source: Br J Cancer. 2020 Jul 9;123(7):1089–100. doi: 10.1038/s41416-020-0956-x (PMC7525450; doi:10.1038/s41416-020-0956-x)
Supplement: Supplementary file 15 — Table S3 [file 41416_2020_956_MOESM15_ESM.docx]

| **Gene** | ***p*-value** |
| --- | --- |
| Clu | 0.00242 |
| S100a8 | 0.00428 |
| Tgfb3 | 0.00428 |
| Tlr2 | 0.00724 |
| Map3k7 | 0.00939 |
| Ulbp1 | 0.0123 |
| Cd33 | 0.0155 |
| Ccl6 | 0.0158 |
| Msln | 0.0164 |
| Ltbr | 0.018 |
| C5ar1 | 0.00275 |

**Table S3**

Significantly altered NanoString immune genes at the “late” tumour regrowth to ≥400mm^3^ time point following 3x5Gy radiotherapy treatment of TRAMP-C1 flank tumour allografts.
